# Comparison of Periodontal Bacteria of Edo and Modern Periods Using Novel Diagnostic Approach for Periodontitis With Micro-CT

**DOI:** 10.3389/fcimb.2021.723821

**Published:** 2021-09-20

**Authors:** Takahiko Shiba, Keiji Komatsu, Takeaki Sudo, Rikai Sawafuji, Aiko Saso, Shintaroh Ueda, Takayasu Watanabe, Takashi Nemoto, Chihiro Kano, Takahiko Nagai, Yujin Ohsugi, Sayaka Katagiri, Yasuo Takeuchi, Hiroaki Kobayashi, Takanori Iwata

**Affiliations:** ^1^Department of Periodontology, Graduate School of Medical and Dental Sciences, Tokyo Medical and Dental University, Tokyo, Japan; ^2^Department of Lifetime Oral Health Care Sciences, Graduate School of Medical and Dental Sciences, Tokyo Medical and Dental University, Tokyo, Japan; ^3^Institute of Education, Tokyo Medical and Dental University, Tokyo, Japan; ^4^Department of Evolutionary Studies of Biosystems, The Graduate University for Advanced Studies (SOKENDAI), Kanagawa, Japan; ^5^Department of Physical Therapy, Faculty of Rehabilitation, Niigata University of Health and Welfare, Niigata, Japan; ^6^Department of Biological Sciences, Graduate School of Science, The University of Tokyo, Tokyo, Japan; ^7^Department of Legal Medicine, Toho University School of Medicine, Tokyo, Japan; ^8^Department of Chemistry, Nihon University School of Dentistry, Tokyo, Japan

**Keywords:** periodontitis, periodontal microbiome, EDO, 16S rDNA sequencing, ancient skeletons

## Abstract

Ancient dental calculus, formed from dental plaque, is a rich source of ancient DNA and can provide information regarding the food and oral microbiology at that time. Genomic analysis of dental calculus from Neanderthals has revealed the difference in bacterial composition of oral microbiome between Neanderthals and modern humans. There are few reports investigating whether the pathogenic bacteria of periodontitis, a polymicrobial disease induced in response to the accumulation of dental plaque, were different between ancient and modern humans. This study aimed to compare the bacterial composition of the oral microbiome in ancient and modern human samples and to investigate whether lifestyle differences depending on the era have altered the bacterial composition of the oral microbiome and the causative bacteria of periodontitis. Additionally, we introduce a novel diagnostic approach for periodontitis in ancient skeletons using micro-computed tomography. Ancient 16S rDNA sequences were obtained from 12 samples at the Unko-in site (18th-19th century) of the Edo era (1603–1867), a characteristic period in Japan when immigrants were not accepted. Furthermore, modern 16S rDNA data from 53 samples were obtained from a database to compare the modern and ancient microbiome. The microbial co-occurrence network was analyzed based on 16S rDNA read abundance. *Eubacterium* species, *Mollicutes* species, and *Treponema socranskii* were the core species in the Edo co-occurrence network. The co-occurrence relationship between *Actinomyces oricola* and *Eggerthella lenta* appeared to have played a key role in causing periodontitis in the Edo era. However, *Porphyromonas gingivalis, Fusobacterium nucleatum* subsp. *vincentii*, and *Prevotella pleuritidis* were the core and highly abundant species in the co-occurrence network of modern samples. These results suggest the possibility of differences in the pathogens causing periodontitis during different eras in history.

## Introduction

There is tremendous interest in studying the evolutionary ecology of the microbiome through the comparative analysis of both ancient and modern forms (Warinner et al., 2015). Recently, calcified dental plaque that had turned into dental calculus in ancient human skeletons was identified as an informative source of ancient human-associated microbial DNA (Adler et al., 2013; Warinner et al., 2014; Weyrich et al., 2017). Genomic analysis of calculus derived from Neanderthals and other ancient humans revealed the diet of that era and how the bacterial composition in ancient humans differed from that of modern humans (Adler et al., 2013; Warinner et al., 2014; Lloyd-Price et al., 2017; Weyrich et al., 2017; Velsko et al., 2019). Furthermore, Adler et al. (2013) revealed that the microbiome reflected changes in Neolithic hunting and harvesting of medieval crops.

Periodontitis is a disease triggered by dental plaque accumulation, causing an inflammatory reaction and bone destruction (Kinane et al., 2017). Many environmental factors, such as smoking habit, diabetes, and host genetics, are known risk factors for periodontitis. Periodontitis is the most common cause of tooth loss among modern adults (Burt, 2005; Petersen et al., 2005; Pihlstrom et al., 2005; Darveau, 2010; Duran-Pinedo et al., 2014) and was detected in the Neanderthals as well (Lozano et al., 2013). In Japan, there have been a few reports of periodontal disease in the past era (Fujita, 2012; Saso and Kondo, 2019). Wooden denture plates, similar to plate dentures at present day, were already present during the Edo era (Edwin L. Cooper, 2004), suggesting that people in the Edo era also experienced tooth loss due to mainly dental caries or periodontal disease (Oyamada et al., 2017). Some studies discussed the diagnosis of periodontal disease in the ancient skeleton (Clarke et al., 1986; Kerr, 1988; Kerr, 1991; Larsen, 1995; Lavigne and Molto, 1995). However, so far, there is no general consensus for diagnostic criteria of periodontal disease in ancient skeletons.

In modern times, there are various theories regarding the bacteriological etiology of periodontal disease, including theories that the “keystone pathogen,” specific low-abundance bacteria can change a healthy microbiome into a dysbiotic state (Yost et al., 2015; Lamont et al., 2018) and that the red complex, which is composed of three pathogens, *P. gingivalis*, *Tannerella forsythia*, and *Treponema denticola*, is frequently observed in periodontitis (Socransky et al., 1998). Additionally, Hajishengallis et al. reported that *P. gingivalis* could be a keystone pathogen of the periodontal disease, and that the dysbiotic biofilm altered by *P. gingivalis* will contribute to periodontal disease process (Hajishengallis et al., 2012). Since the development of a high-throughput sequencer, it has become possible to comprehensively identify the bacterial composition of dental plaque in periodontitis by calculating the number of sequencing reads that align with the reference genomes (Maruyama et al., 2014; Shiba et al., 2016; Komatsu et al., 2020). There have been many studies analysing ancient bacterial genomes from Europe and America. Warinner et al. confirmed that the red complex has long been associated with periodontal disease in German skeletons carbon dated to c. 950–1200 CE (Warinner et al., 2014). However, the microbiome involved in the risk of disease development is regional (Karlsson et al., 2013). There are a few reports regarding bacterial genomic analysis for the Japanese Edo era (Eisenhofer et al., 2020; Sawafuji et al., 2020), and no studies discuss the oral microbiome related to periodontal disease using ancient calculus.

In this study, we introduced a novel diagnostic approach for periodontitis in an ancient skeleton by simultaneous visual inspection and micro-computed tomography (micro-CT). Additionally, we performed 16S rDNA sequencing with a next-generation sequencer to obtain information regarding bacterial genomes contained in the calculus derived from ancient Japanese alveolar bones of the Edo era. Furthermore, the purpose of this study was to explore timeless or time-specific bacteria groups involved in periodontitis by comparing Edo era and modern time.

## Material and Methods

### Morphological Examinations

**Figure 1:**
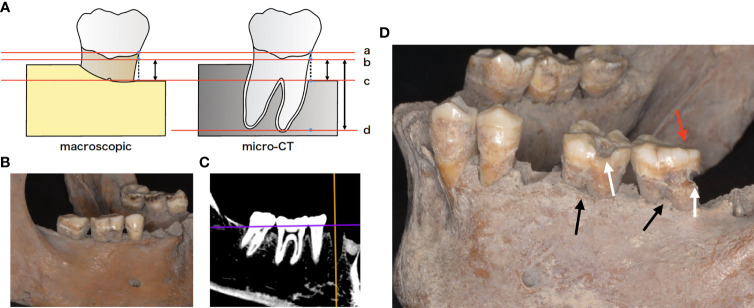
**(A)** Schematic representation of the morphologic examination. Bone resorption was defined as the distance from virtual alveolar crest, which was obtained by subtracting 1 mm from the cemento-enamel junction to the bottom of the nearest bone defect (b, c). Bone resorption was measured using both periodontal probe and micro-computed tomography (micro-CT). Root length was defined as the distance from virtual alveolar crest to the apical end of the root (a–d). Bone resorption ratio was calculated as (b-c)/(b-d). a cement-enamel junction (CEJ), b virtual alveolar crest, c bottom of bone defect (bone level), d: apical end of the root. **(B)** Mandibular skeleton (sample F24). **(C)** micro-CT image of the same bone. **(D)** Tooth morphologic characteristics. White arrows indicate cavities, while black arrows indicate furcation involvement. Red arrows indicate occlusal attrition.

The Unko-in site is the former graveyard of the Unko-in temple in Fukagawa, Tokyo; in 1955, more than 200 skeletons were excavated from this site. The skeletons were determined to be from the Edo era (18th–19th century). The skeletons are housed at the University Museum, University of Tokyo (UMUT). Ethical review and approval were not required for this study because we used only the museum specimens deposited in scientific collections for this study. Morphological examinations were performed to evaluate the periodontal status of the ancient skeletons by dentists in the department of periodontology at Tokyo Medical and Dental University (TMDU). Instead of measuring the probing depth or clinical attachment level, which are used to evaluate periodontal inflammation and destruction in routine clinical practice, vertical bone loss—defined as the length from 1 mm below the cemento-enamel junction to the most apical extent of the alveolar bone—was measured at six sites (mesiobuccal, midbuccal, distobuccal, mesiolingual, midlingual, and distolingual) on each tooth with a periodontal probe (UNC-15 University of North Carolina 15, Hu-Friedy^®^, Chicago, IL, USA) to calculate the average bone loss per sample. Additionally, micro-CT (TESCO Corporation, Tokyo, Japan) was used to calculate the vertical bone resorption ratio by measuring both the mesial and distal bone levels and root length in the mid-tooth section (**Figure 1**). All teeth in the 12 skeletons were examined for attrition, furcation involvement, cavities, and torus.

### Sample Collection and DNA Extraction

Supragingival and subgingival calculus were collected and combined from the teeth of 12 adult human skeletons at UMUT. Calculus from each individual skeleton was collected separately in 1.5 mL DNA LoBind tubes (Eppendorf, Hamburg, Germany). As controls, the genomic DNA samples of the soil near the mandibular foramen of the skeletons and sterilized water were subjected to the same analysis as the calculus. Masks, nitrile gloves, hairnets, and laboratory coats were worn throughout the process. DNA extraction was performed in a dedicated ancient DNA laboratory at Tokyo University. To avoid contamination, DNA extraction was performed as described in a previous report (Sawafuji et al., 2020).

### Library Preparation and 16S rDNA Sequencing

The 16S rDNA sequencing was performed by sequencing 2 × 300 bp paired-end reads targeting the hypervariable region of V4 using the MiSeq platform (Illumina, San Diego, CA, USA) (Santiago-Rodriguez et al., 2019). Library preparation was conducted according to the Illumina 16S sample preparation guide (16S Sample Preparation Guide, Illumina) with appropriate precautions, such as the utilization of UV-irradiated clean bench, 80% ethanol, filtered pipettes, and reagents used for only ancient samples in the normal laboratory at TMDU. The DNA sample of the V4 region was amplified with HiFi hot fidelity primer (New England Biolabs, Ipswich, MA, USA) and with forward 515 F (5-TCGTCGGCAGCGTCAGATGTGTATAAGAGACAGGTGCCAGCMGCCGCGGTAA-3) and reverse primer 806 R (5-GTCTCGTGGGCTCGGAGATGTGTATAAGAGACAGGGACTACHVGGGTWTCTAAT-3) (Cano et al., 2014; Yang et al., 2016; Qu et al., 2017; Santiago-Rodriguez et al., 2019). Negative controls were also included in the library preparation. The polymerase chain reaction (PCR) cycling conditions were as follows: 95°C for 3 min; 35 cycles of 95°C for 30 s, 55°C for 30 s, and 72°C for 30 s; and 72°C for 5 min. The amplified products were assessed for purity using an Agilent 2100 Bioanalyzer (Agilent Technologies, Santa Clara, CA, USA) and were further purified using the ProNex Size-Selective Purification System (Promega, Madison, WI, USA). These purified products were attached to Illumina sequencing adapters using the Nextera XT index primer and purified with the ProNex^®^ Size-Selective Purification System (Promega). The quantity of each purified sample was evaluated by quantitative PCR (qPCR) using the KAPA Library Quantification Kit (KAPA Biosystems, Woburn, MA, USA), and the quantity of each sample was normalized to maintain a concentration same as that of the pooled library. The final library was contained in the PhiX Control library (v3) (Illumina) to support cluster densities, while sequencing data of 16S rDNA were obtained using the MiSeq V3 reagent kit (Illumina). MiSeq sequencing of the 16S rRNA gene was conducted at Tokyo Dental College.

### Data Analysis

The 16S rDNA sequencing reads were processed using the Illinois Mayo Taxon Organization from the RNA Dataset Operations pipeline (IM-TORNADO) (Jeraldo et al., 2014) for 300 bp reads that merges paired-end reads into a single multiple alignment. Reads with a minimum length of 187 bases passed quality control. The forward and reverse reads were trimmed to 200 and 250 bases, respectively. The similarity of operational taxonomic unit (OTU) binning was 97%. Each OTU was assigned at the species level using the Human Oral Microbial Database (v15.11) (Chen et al., 2010) with 97% sequence identity. The singletons were removed. OTUs identified in the negative controls were removed from the samples because they were considered to be contaminated with modern DNA (Weyrich et al., 2017). All abundance values were normalized by conversion to reads per million values. Rarefaction curve, number of OTUs, and the Shannon index were calculated as α diversity. The rarefaction single command in Mothur v1.33.3 was used to generate the rarefaction curve (Schloss et al., 2009). Principal coordinate analysis (PCoA) plots were visualized using R v.3.3.2 software. Dissimilarity values (1-Spearman correlation) were clustered using the average linkage method. Co-occurrence coefficients were calculated based on adjusted read abundances using the sparse correlations for compositional data algorithm (SparCC) program (Milici et al., 2016). For comparison with the subgingival microbiome of the modern Japanese population, we obtained FASTQ files of V3–V4 amplicon sequence data of subgingival plaque samples from DDBJ under accession numbers DRA008582 (Iwauchi et al., 2019) and DRA010104 (Komatsu et al., 2020). Briefly, DRA010104 was generated in exactly similar conditions as the Edo sample in this paper. In contrast, DRA008582 is genomic data generated by amplifying the V3–V4 region with the following parameters: preheating at 94°C for 3 min; 30 cycles of denaturation at 94°C for 30 s, annealing at 50°C for 30 s, and extension at 72°C for 30 s; and a terminal extension at 72°C for 5 min. Network structures were constructed using two species taxa with a positive correlation of relative read abundance with SparCC values ≥ 0.3 (Shiba et al., 2016). Co-occurrence patterns were drawn using a network structure wherein each taxon and co-occurrence were indicated by a node and edge, respectively, for all taxon pairs with a positive correlation. Networks were visualized using Cytoscape software v.2.860 (Smoot et al., 2011).

### Statistical Analysis

Wilcoxon’s rank-sum test was performed to test for significant differences in each taxon between the groups. The number of OTUs and Shannon index were compared using a two-tailed t-test. *P*-values < 0.05 were considered statistically significant. Benjamin and Hochberg’s false discovery rate was applied for multiple testing, and *q* < 0.1 was considered statistically significant. An analysis of similarity (ANOSIM) was used to test the significance of dissimilarity between the two groups by applying adjusted read abundance.

## Results

### Clinical Characteristics

Samples with bone loss >4 mm or bone loss ratio >10% were diagnosed with periodontitis, and 5 out of 12 samples were affected by periodontitis. Caries and attrition were observed in 7 and 10 samples, respectively; non-carious cervical lesions (NCCL) and torus were not observed in any of the samples (**Table 1**) (for further details about each sample, see **Supplementary Figures**). Five samples with periodontitis had an average bone loss of 4.05 ± 0.88 mm (mean ± SD) and a mean bone loss ratio of 21.88 ± 9.44%, while 7 samples without periodontitis had an average bone loss of 2.73 ± 0.43 mm and a mean bone loss ratio of 5.32 ± 2.75%. Clinical characteristics of the modern subjects are shown in **Supplementary Table 1**.

**Table 1:** Morphological examination of each individual skeleton.

Sample type: Periodontitis								
Sample No.	EP1	EP2	EP3	EP4		EP5		Mean/Prevalence
Sex	Female	Female	Male	Male		Male		–
Average bone loss (mm)	3.55 ± 1.21	4.94 ± 1.84	2.86 ± 0.72	5.22 ± 1.78		3.69 ± 0.88		4.05 ± 0.88
Average ratio of bone resorption (%)	17.75 ± 9.71	20.76 ± 8.35	18.67 ± 13.10	39.93 ± 16.75		12.31 ± 10.33		21.88 ± 9.44
Attrition	+	+	+	+		+		100%
Furcation involvement	+	+	+	+		+		100%
Cavity	+	+	–	+		–		60%
Torus	–	–	–	–		–		0%
Non caries cervical lesion	–	–	–	–		–		0%
Periodontitis	+	+	+	+		+		100%
**Sample type: Healthy**								
**Sample No**.	**EH1**	**EH2**	**EH3**	**EH4**	**EH5**	**EH6**	**EH7**	**Mean/Prevalence**
Sex	Male	Female	Male	Male	Female	Female	Female	–
Average bone loss (mm)	3.70 ± 1.70	2.98 ± 0.78	2.61 ± 1.15	2.30 ± 0.75	2.53 ± 0.56	2.61 ± 1.15	2.42 ± 0.67	2.73 ± 0.43
Average ratio of bone resorption (%)	7.18 ± 11.95	5.53 ± 11.16	2.83 ± 5.97	8.69 ± 13.21	5.84 ± 5.95	0	7.23 ± 6.03	5.32 ± 2.75
Attrition	+	+	+	+	+	–	–	71.42%
Furcation involvement	+	+	+	+	+	+	–	85.71%
Cavity	–	–	–	+	+	+	+	57.14%
Torus	–	–	–	–	–	–	–	0%
Non caries cervical resion	–	–	–	–	–	–	–	0%
Periodontitis	–	–	–	–	–	–	–	0%

Values are presented as mean ± standard deviation.

Periodontitis was defined as one which has either more than 4mm of average bone resorption or more than 10% of average ratio of bone resorption.

### Summary of Sequence Reads and Analysis of Diversity

**Figure 2:**
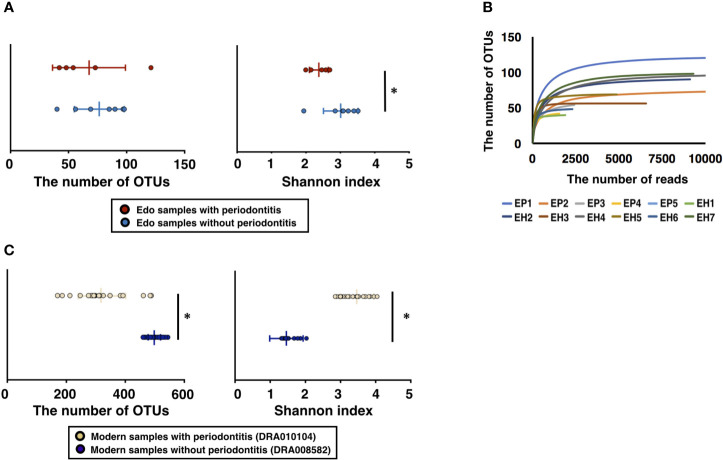
Evaluation of bacterial diversity of ancient Edo sample based on 16S rDNA sequences. **(A)** The number of operational taxonomic units (OTUs) and Shannon index of Edo samples. **(B)** Rarefaction curve of Edo samples. **(C)** The number of OTUs and Shannon index of modern samples. *P < 0.05.

A total of 750,940 ancient sequence reads were obtained from 16S rDNA sequencing. The average number of reads per sample was 62578.33 ± 13249.49. After analysis of the IM-TORNADO pipeline, the remaining ancient reads per sample were 31020.75 ± 7372.38 (**Supplementary Table 2**). A total of 477, 192, and 17 OTUs were detected in the ancient samples, soil, and water, respectively. The OTUs detected in soil and water were considered the contamination from modern times and removed from Edo samples. After removing contamination caused by modern DNA, the number of remaining reads and OTUs was 6847.16 ± 4179.48 and 72.75 ± 24.62, respectively, in all Edo samples (**Supplementary Table 2**). The number of OTUs in the Edo samples with periodontitis was 67.60 ± 28.65, and the Shannon index was 2.37 ± 0.26. The number of OTUs using the Edo samples without periodontitis was 76.43 ± 20.51, and the Shannon index was 3.01 ± 0.48 (**Supplementary Table 3**). Although there was no significant difference in the number of OTUs between the Edo samples with and without periodontitis, the Shannon index of the Edo samples without periodontitis was significantly higher than that of Edo samples with periodontitis (**Figure 2A**). The rarefaction curve showed that the obtained ancient reads were sufficient to examine the comprehensive bacterial composition, as the obtained OTUs reached saturation (**Figure 2B**); 11692.46 ± 2028.79 and 24311.00 ± 17013.88 reads were used for taxonomic analysis in the IM-TORNADO pipeline using modern samples with and without periodontitis, respectively (**Supplementary Table 4**). The number of OTUs using the modern samples with periodontitis was 317.08 ± 81.05, and the Shannon index was 3.47 ± 0.48 (**Figure 2C** and **Supplementary Table 5**). The number of OTUs using modern samples without periodontitis was 493.93 ± 23.39, and the Shannon index was 1.45 ± 0.16 (**Figure 2C** and **Supplementary Table 5**).

### Evaluation of Bacterial Composition at the Phylum Level Based on 16S rDNA Sequencing

**Figure 3:**
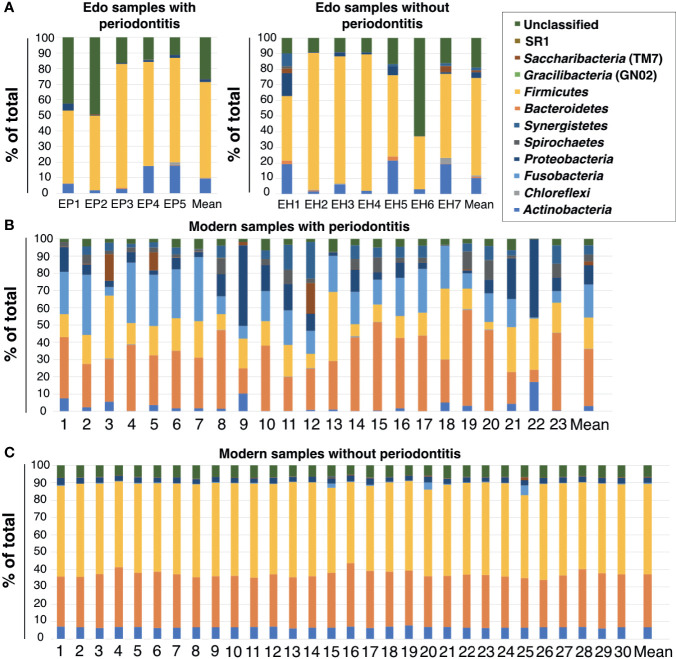
Percentage compositions of the rank of phylum. **(A)** Edo samples, **(B)** modern samples with periodontitis, and **(C)** modern samples without periodontitis.

Eight bacterial phyla were detected in the ancient dental calculus: *Firmicutes, Actinobacteria, Proteobacteria, Synergistetes, Saccharibacteria* (TM7), *Chloroflexi, Bacteroidetes*, and *Spirochaetes* (**Figure 3A** and **Supplementary Table 6**). *Firmicutes* was the most dominant phylum in Edo samples with and without periodontitis, followed by *Actinobacteria* and *Proteobacteria*, except for the unclassified phylum. The ANOSIM evaluation revealed similar bacterial compositions at the phylum level for Edo samples with and without periodontitis (*R* = 0.9417 and *P* = 0.001).

The most abundant phylum in the modern samples with periodontitis was *Bacteroidetes*, followed by *Fusobacteria* and *Firmicutes* (**Figure 3B** and **Supplementary Table 7**). The most abundant phylum in the modern samples without periodontitis was *Firmicutes*, followed by *Bacteroidetes* and *Actinobacteria* (**Figure 3C** and **Supplementary Table 7**). There were no major differences in the phyla between the Edo samples with and without periodontitis. Although both *Fusobacteria* and SR1 were detected in the modern samples with and without periodontitis, they were not observed in Edo samples. *Gracilibacteria* (GN02) is a specific phylum in modern samples with periodontitis. The ANOSIM evaluation revealed that, at the phylum level, the bacterial compositions of Edo and modern samples with periodontitis were dissimilar (*R* = 0.9417 and *P* = 0.0010).

### Evaluation of Bacterial Composition at the Class Level Based on 16S rDNA Sequencing

*Clostridia* were detected as being the most abundant in Edo samples with and without periodontitis, excluding the unclassified class, followed by *Actinobacteria* (**Supplementary Table 8**). Comparing Edo samples with periodontitis to those without periodontitis, *Flavobacteriia* was specific to Edo samples with periodontitis. *Epsilonproteobacteria, Erysipelotrichia*, and *Sphingobacteriia* were specific to Edo samples without periodontitis. However, the ANOSIM evaluation revealed similar bacterial compositions at the class level for Edo samples with and without periodontitis (*R* = -0.0065 and *P* = 0.4300).

**Figure 4:**
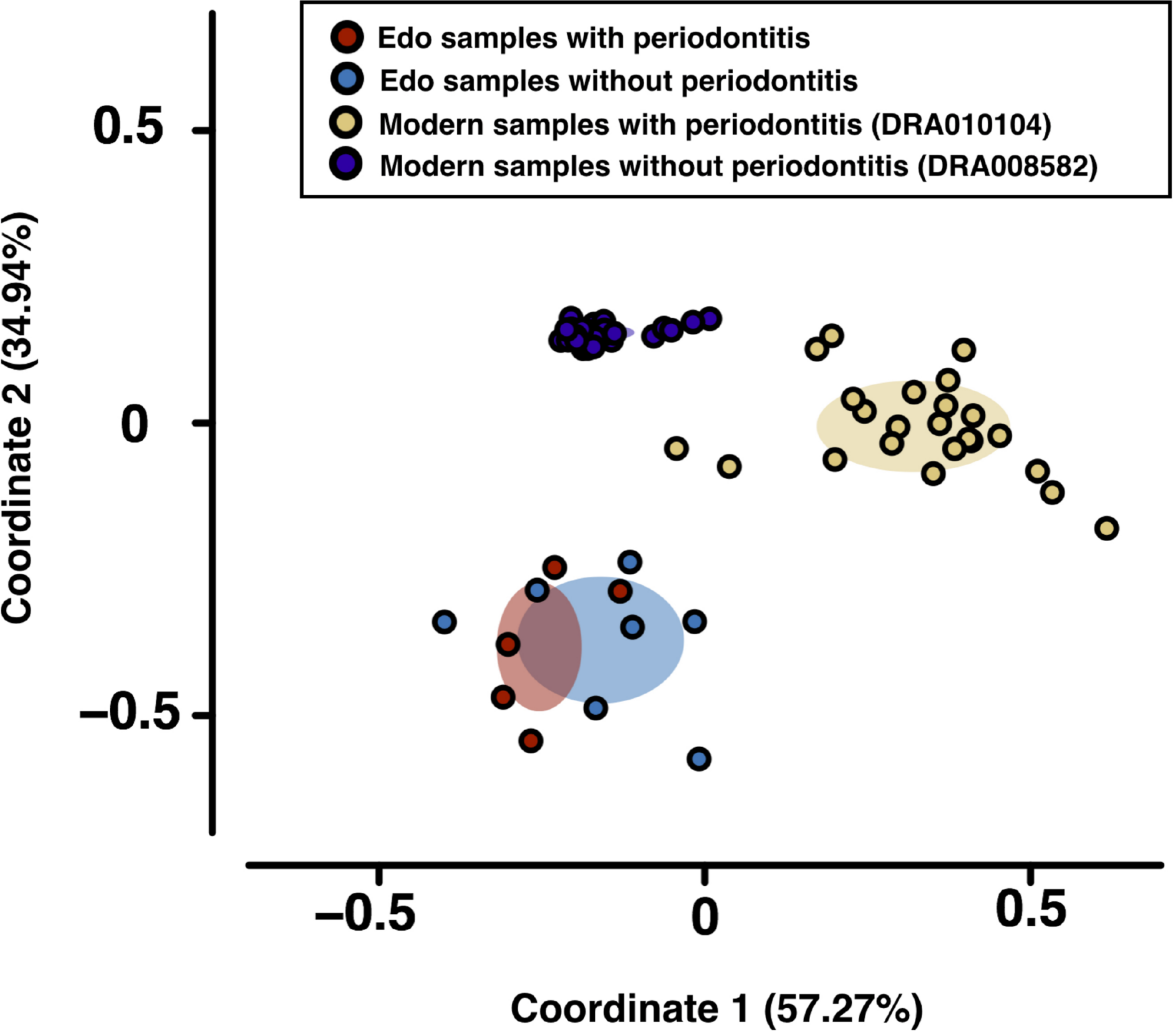
Principal coordinate analysis (PCoA) was conducted for the dissimilarity matrix value of 1—Spearman**’**s coefficient. PCoA was used to evaluate bacterial similarity for comparison between Edo and modern microbiomes according to class.

*Bacteroidia* and *Clostridia* were most abundant in modern samples with and without periodontitis, respectively (**Supplementary Table 9**). PCoA was used to evaluate bacterial similarity for comparison between Edo and modern microbiomes (**Figure 4**). Although Edo samples with and without periodontitis had similar bacterial composition, modern and Edo samples with periodontitis had dissimilar bacterial composition at the class level. ANOSIM revealed dissimilarity between the modern and Edo samples with periodontitis (*R* = 0.9118 and *P* = 0.0001).

### Evaluation of Bacterial Composition at the Genus Level Based on 16S rDNA Sequencing

**Figure 5:**
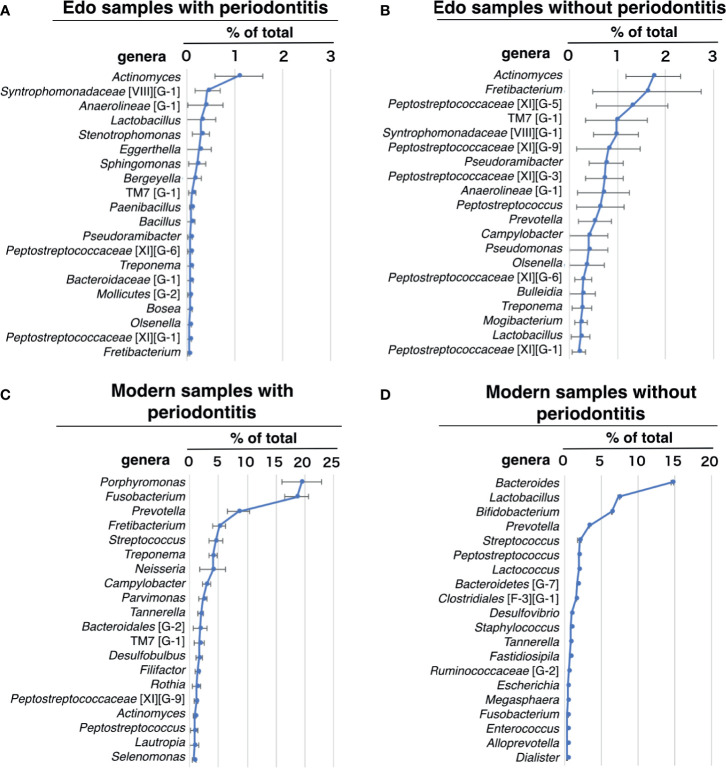
Percentage compositions of genus. **(A)** Edo sample with periodontitis, **(B)** Edo sample without periodontitis, **(C)** modern samples with periodontitis, and **(D)** modern sample without periodontitis. The bars show mean ± SE relative abundances.

*Actinomyces*, which was detected in all Edo samples, was the most dominant genus in the Edo samples, both with and without periodontitis (**Figures 5A, B** and **Supplementary Table 10**). *Syntrophomonadaceae* [VIII][G-1] was the second most dominant genus, followed by *Anaerolineae* (G-1) in Edo samples with periodontitis (**Figure 5A** and **Supplementary Table 10**). *Fretibacterium* was the second most dominant genus, followed by *Peptostreptococcaceae* [XI][G-5] in Edo samples without periodontitis (**Figure 5B** and **Supplementary Table 10**). In Edo samples with and without periodontitis, 37 and 24 genera, respectively, were detected—excluding the unclassified genera. Among these, 22 genera were common between the Edo samples with and without periodontitis. The ANOSIM evaluation revealed similar bacterial compositions at the genus level for Edo samples with and without periodontitis (*R* = -0.0470 and *P* = 0.5980). In modern samples with and without periodontitis, 93 and 74 genera, respectively, were detected—excluding the unclassified genera. Among these, 54 genera were common between the modern samples with and without periodontitis. The most abundant genus in the modern samples with periodontitis was *Porphyromonas*, followed by *Fusobacterium* and *Prevotella* (**Figure 5C** and **Supplementary Table 11**). The most abundant genus in the modern samples without periodontitis was *Bacteroides*, followed by *Lactobacillus* and *Bifidobacterium* (**Figure 5D** and **Supplementary Table 11**). On comparing Edo and modern samples with periodontitis, *Syntrophomonadaceae* [VIII][G-1], *Lactobacillus*, *Stenotrophomonas, Eggerthella, Bergeyella, Paenibacillus, Bosea*, and *Clostridiales* [F-3][G-1] were found to be specific to Edo samples with periodontitis. Contrarily, 77 genera were detected only in modern samples with periodontitis. The ANOSIM evaluation revealed that the bacterial composition at the genus level for Edo and modern samples with periodontitis was dissimilar (*R* = 0.9753 and *P* = 0.0010).

### Evaluation of Bacterial Composition at the Species Level Based on 16S rDNA Sequencing

**Figure 6:**
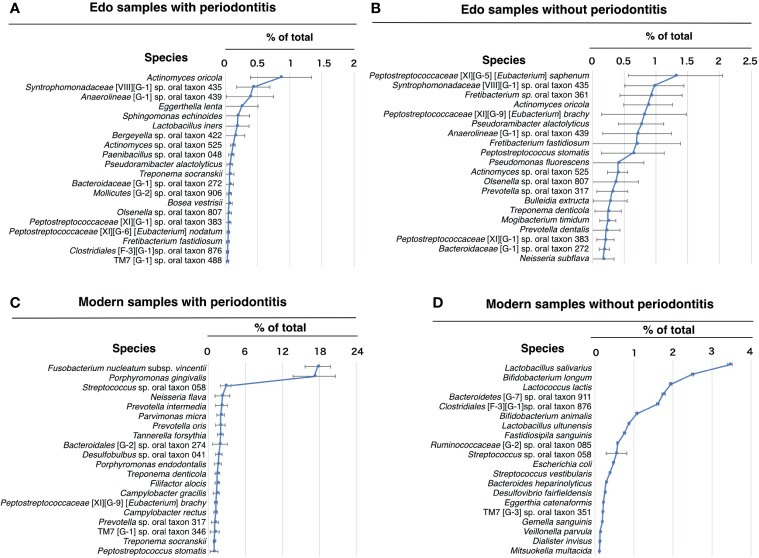
Percentage compositions according to species. **(A)** Edo sample with periodontitis, **(B)** Edo sample without periodontitis, **(C)** modern samples with periodontitis, and **(D)** modern sample without periodontitis. The bars show mean ± SE relative abundances.

**Figure 7:**
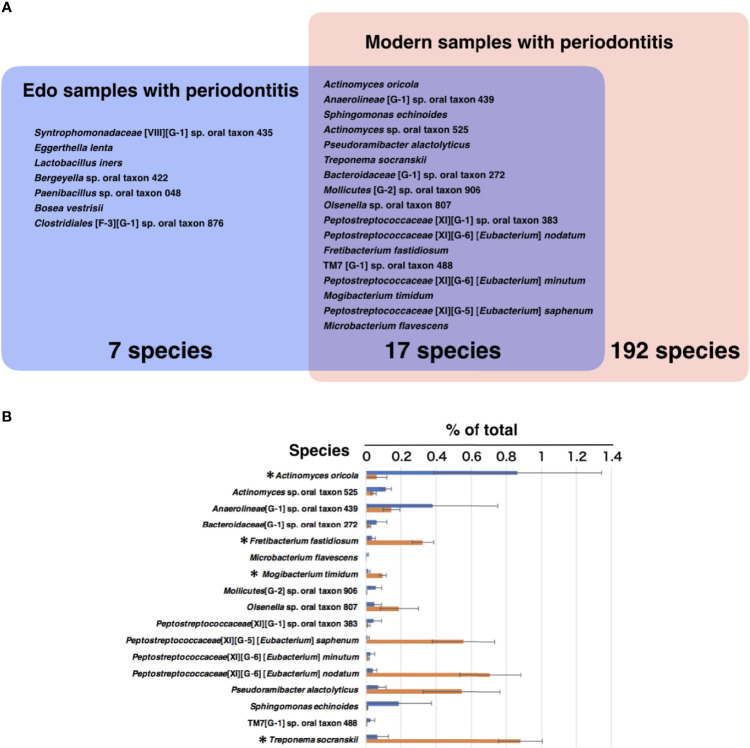
The core microbiome of Edo and modern samples with periodontitis. **(A)** The model includes the species detected in Edo sample with periodontitis (blue), modern sample with periodontitis (red), and both sites (purple); red text indicates species existing in both co-occurrence networks. **(B)** The mean relative abundances of common species between Edo and modern samples with periodontitis. The bars show mean ± SE relative abundances.

The most abundant species in the Edo samples with periodontitis was *Actinomyces oricola*, which was the only species detected in all the Edo samples with periodontitis. The second most abundant species was *Syntrophomonadaceae* [VIII][G-1] sp. oral taxon 435, followed by *Anaerolineae* [G-1] sp. oral taxon 439 (**Figure 6A** and **Supplementary Table 12**). The most abundant species in the Edo samples without periodontitis was *Peptostreptococcaceae* [XI][G-5] [*Eubacterium*] *saphenum*, followed by *Syntrophomonadaceae* [VIII][G-1] sp. oral taxon 435 and *Fretibacterium* sp. oral taxon 361 (**Figure 6B** and **Supplementary Table 12**). ANOSIM evaluation revealed similar bacterial compositions at the species level for Edo samples with and without periodontitis (*R* = 0.0000 and *P* = 0.5640). The most abundant species in the modern samples with periodontitis was *Fusobacterium nucleatum* subsp. *vincentii*, followed by *P. gingivalis* and *Streptococcus* sp. oral taxon 058 (**Figure 6C** and **Supplementary Table 13**). However, the most abundant species in the modern sample without periodontitis was *Lactobacillus salivarius*, followed by *Bifidobacterium longum* and *Lactococcus lactis* (**Figure 6D** and **Supplementary Table 13**). Seventeen species that were common to Edo and modern samples with periodontitis were detected, while the number of species specific to Edo and modern samples with periodontitis were 7 and 192, respectively (**Figure 7A**). *A. oricola* was the only species that was significantly more abundant in Edo samples with periodontitis than in the modern samples with periodontitis among the 17 species that were common to the Edo era and the modern times (**Figures 7A, B**). In contrast, *Fretibacterium fastidiosum*, *Mogibacterium timidum*, and *Treponema socranskii* were more frequent in the modern samples with periodontitis than in the Edo samples with periodontitis. ANOSIM showed that the composition of species was not similar for Edo and modern samples with periodontitis (*R* = 0.9132 and *P* = 0.0010).

### Comparison of Co-Occurrence Networks Between Edo and Modern Samples With Periodontitis

**Figure 8:**
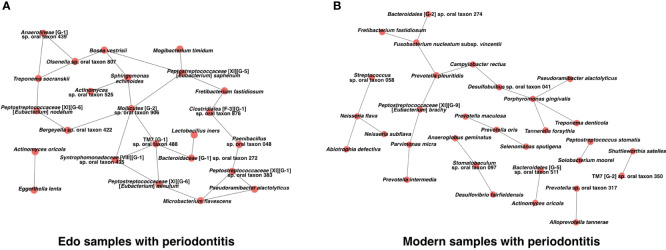
Co-occurrence networks based on species in **(A)** Edo sample with periodontitis and **(B)** modern sample with periodontitis.

We constructed a co-occurrence network structure to identify the core species in the microbiome. Two co-occurring species are indicated by nodes and connected by edges. For Edo samples with periodontitis, the total numbers of nodes and edges were 24 and 29 at the species level, respectively (**Figure 8A** and **Supplementary Table 14**). Co-occurrence networks of both Edo and modern samples with periodontitis shared *Fretibacterium fastidiosum*, *Neisseria subflava*, *Peptostreptococcaceae* [XI][G-9] [*Eubacterium*] *brachy*, *Prevotella* sp. oral taxon 317, *Pseudoramibacter alactolyticus*, *T. denticola*, *A. oricola*, and *Peptostreptococcus stomatis*. The number of species that were connected with three or more nodes in the co-occurrence network of Edo samples with periodontitis was 11. Particularly, *Mollicutes* [G-2] sp. oral taxon 906, *Peptostreptococcaceae* [XI][G-5] [*Eubacterium*] *saphenum*, and TM7 [G-1] sp. oral taxon 488 were the core species that were connected to more than four nodes in the network. For modern samples with periodontitis, the total number of nodes and edges were 31 and 23 at the species level, respectively (**Figure 8B** and **Supplementary Table 15**). Four species were connected to three or more nodes in the co-occurrence network of modern samples with periodontitis. *P. gingivalis* was the core species that was connected to *P. alactolyticus*, *T. denticola*, *T. forsythia*, and *Desulfobulbus* sp. oral taxon 041. The values of network density were 0.105 and 0.049 for the Edo and modern samples with periodontitis, respectively.

## Discussion

The purpose of this study was to comprehensively analyze the bacterial composition and co-occurrence network of the microbiome in the Edo era and elucidate the bacterial species involved in periodontitis in the Edo era and modern samples. Additionally, this study revealed characteristic intraoral findings in Edo samples and introduced novel morphological examinations to evaluate the periodontal status of ancient skeletons.

The presence or absence of periodontal disease in ancient skeletons has been evaluated based on antemortem tooth loss (AMTL) (Clarke et al., 1986; Lavigne and Molto, 1995). Larsen (1995) argued that AMTL has a strong relationship with periodontal disease in archaeology. However, it is difficult to identify the cause of AMTL in many cases, such as dental caries, periodontitis, and tooth fracture. Therefore, the distance from the cemento-enamel junction (CEJ) to the alveolar crest (AC) and morphological assessment of inflammation of the interdental septum have often been used for the diagnosis of periodontal disease (Clarke et al., 1986; Kerr, 1988; Kerr, 1991; Lavigne and Molto, 1995). The CEJ-AC distance measures the root exposure to assess the degree of alveolar resorption (Clarke et al., 1986). Root exposure with gingival recession is affected by the lack of alveolar bone at the site due to fenestration and dehiscence of alveolar bone, abnormal tooth position, direction of eruption of the tooth, and the tooth shape, except for periodontitis (Kassab and Cohen, 2003). The morphological assessment of inflammation of the interdental septum involves measurement of the septal form characteristic, and it is difficult to characterize the form due to scratches during excavation of the skeleton or preservation under an inadequate environment. It is also difficult to measure the depth and angular degree of deep intrabony defect in category 5, proposed by Kerr (Kerr, 1988; Kerr, 1991). Recently, Warinner used the degree of bone resorption relative to root length for assessing periodontal destruction in ancient skeletons (Warinner et al., 2014). To grasp the root length, the tooth has to be removed from the ancient skeleton. In recent years, CBCT has seen remarkable development and has become essential in current dental treatment to understand the morphology of bone resorption due to periodontal disease (Braun et al., 2014). Additionally, the root length can be accurately ascertained without removing the tooth from the ancient skeleton using CBCT. Therefore, we applied CBCT for the periodontal examination of the ancient skeleton.

In this study, periodontal disease was diagnosed in skeletons from the Edo era with a prevalence rate of approximately 42% (5 of the 12 skeletons). The second term (2013 to 2022) of the National Health Promotion Movement in the 21st century (Health Japan 21) reported a prevalence of periodontal disease among Japanese people in their forties to be 37.3% in 2005, with a targeted reduction to 25% by 2022 (Ministry of Health, 2012). Contrary to expectations, the prevalence of periodontal disease among individuals in the Edo era and modern population was similar. NCCL was not observed in the Edo samples used in this study. At present, occlusion is no longer considered the cause of NCCL, and only brushing has been verified in an experimental model (Fan and Caton, 2018). During the Edo era, brushing techniques and materials were not as advanced as the current ones. A report on DNA analysis of the food remains in human dental calculus from the Edo era in Japan using next-generation sequencing helped detect plant DNA of the genus *Nicotiana* (Sawafuji et al., 2020). The use of tobacco has also been reported in the historical literatures in the Edo period (Kitagawa, 1853; Miyazaki, 1967). Thus, it can be presumed that the people in the Edo era were exposed to the same periodontal disease risk factors as people in modern times.

Eleven phyla were detected in the bacterial composition of the modern samples in this study. In contrast, *Fusobacteria*, SR1, and *Gracilibacteria* (GN02) were detected only in modern samples, and unique phyla were not detected in the Edo samples. Although there were no significant differences between Edo and modern samples at the phylum level, PCoA plots at the class level showed that the bacterial composition of Edo samples was distinctly different from that of modern samples. Interestingly, *Fusobacteria*, which are typical pathogenic bacteria of periodontitis, were never detected in the ancient Edo samples at the phylum level. It is well-known that *F. nucleatum*, which belongs to phylum *Fusobacteria*, plays an important role in oral bacterial colonization. A previous study reported that the calculus of Neanderthals hardly contained *Fusobacteria* (Weyrich et al., 2017). Therefore, we presumed that there might be other key bacteria that contributed to plaque formation before the post-industrial era. The red complex plays a major role in the etiology of periodontal disease in modern humans (Socransky et al., 1998). Kasuga et al. (2000) reported the detection rates of *P. gingivalis*, *T. forsythus*, and *T. denticola* in samples from Japanese adult periodontal lesions to be 75.5%, 69.8%, and 72.6%, respectively. However, these species that were representative periodontal pathogens were not detected in Edo samples. These results are inconsistent with those of previous reports focusing on the ancient calculus microbiome (Adler et al., 2013; Warinner et al., 2014). Microbiome alterations result from various factors, such as changes in diet (Brown et al., 2012) and adoption of lifestyles associated with industrialization (Clemente et al., 2015). Japanese industrialization occurred between the Edo era and modern times. Additionally, the Edo era is recognized for *Sakoku*, which means closed country, with an isolationist foreign policy of the Japanese Tokugawa shogunate in place for a period of 214 years (1639–1854). It resulted in severely limited relations and trade between Japan and other countries; almost all foreigners were barred from entering Japan and common Japanese people were kept from leaving the country (Kazui and Videen, 1982). Since there was no transmission of bacteria from other countries, it is possible that the locals possessed unique and less diverse microbiome. Two hypotheses might arise: one is that periodontitis is not necessarily induced by the red complex, and the other is that potential periodontal pathogens in the Edo era were different from those in modern times.

Considering the bacterial composition at the species level, the number of species common between Edo and modern, Edo specific, and modern specific species were 17, 7, and 192, respectively. *Eggerthella lenta*, a species and genus specific to Edo, is an anaerobic, Gram-positive bacillus commonly found in the human digestive tract (Moore et al., 1971). Occasionally, *E. lenta* can cause life-threatening infections, such as polymicrobial intra-abdominal and anaerobic bloodstream infections (Ugarte-Torres et al., 2018). *Lactobacillus iners*, a species specific to Edo, is a Gram-positive, catalase-negative, facultatively anaerobic rod-shaped bacterium (Falsen et al., 1999). Vaginal microbiome dominated by *L. iners* was associated with increased risk of *Chlamydia trachomatis* infection (van Houdt et al., 2018). *Lactobacillus* species in the oral cavity represent both a significant contributor to dental caries progression and a major reservoir to the gastrointestinal tract (Caufield et al., 2015). There are two possible reasons for this: first, the caries prevalence rate was approximately 58% (7 of the 12 skeletons) in this study. Second, the samples in this study included not only subgingival dental calculus but also supragingival dental calculus. Therefore, the effects of the cariogenic microbiota could not be ignored entirely. Ancient skeletons in which both tooth and dental calculus are preserved in good condition are extremely rare. In addition to the difficulty in obtaining the necessary amount of dental calculus for analysis due to the limited amount of dental calculus, it is difficult to distinguish between supragingival and subgingival dental calculus because there is no soft tissue in the ancient skeletons. Further, the color difference between supragingival and subgingival dental calculus is not apparent because a long time has passed.

The association of Edo specific species with periodontitis has not been reported yet. Focusing on the species common to the Edo era and modern times, 13 species did not show any statistically significant difference between the Edo and modern samples. Therefore, we assumed that these species have associated with periodontitis from the 19th century to the present day in Japan. *A. oricola* in the Edo samples was significantly more abundant than in the modern samples and co-occurred with *E. lenta*. The relationship might have been important for causing periodontitis in the Edo era. Additionally, *Mollicutes* [G-2] sp. oral taxon 906, *Peptostreptococcaceae* [XI][G-1] sp. oral taxon 383, *Peptostreptococcaceae* [XI][G-5] [*Eubacterium*] *saphenum*, *Peptostreptococcaceae* [XI][G-6] [*Eubacterium*] *minutum*, and TM7 [G-1] sp. oral taxon 488 were found to be core species in the co-occurrence network of Edo samples, but not modern samples. *Eubacterium* species, which are asaccharolytic anaerobic Gram-positive rods, produce butyric acids (Uematsu et al., 2003). Recent studies have reported that butyric acid produced by microorganisms has some pathogenicity, such as suppression of human gingival fibroblast proliferation (Jeng et al., 1999) and induction of apoptosis in human or mouse T and B cells (Tse and Williams, 1992; Pöllänen et al., 1997). We presumed that the pathogenicity of these bacteria was higher in the Edo era than in modern times, and the activity of these bacteria could affect the progression of periodontitis in the Edo era. Several studies have reported that TM7 species were significantly enriched in periodontitis in modern times (Liu et al., 2012; Chen et al., 2018). However, the virulence is unknown because TM7 species are unculturable bacteria (Hanada et al., 2014). Similarly, *Mollicutes* were detected in advanced periodontal lesions (Hanada et al., 2014); however, their role is not clear. *T. socranskii* is frequently detected in aggressive and chronic periodontitis in modern times (Takeuchi et al., 2001). It is known that *T. socranskii* produces heat shock proteins implicated in periodontitis in modern times. *T. socranskii* was also a core species in the co-occurrence network of Edo samples, although it was significantly more abundant in the modern samples than in Edo samples. Therefore, *T. socranskii* might have played a more important role in periodontitis during the Edo era than in modern times. All species commonly detected in the Edo era and modern times were present in the co-occurrence network of the Edo samples. In the co-occurrence network of the modern samples, only three species among all species commonly detected in the modern times and Edo era were present, and it seems that these species might not play a key role because they did not have prominent co-occurrence relationships with other species. Therefore, there might be no specific causative bacteria for periodontitis that are timeless, and the causative bacteria for periodontitis might have changed with time and environment. The influx of cultures, especially the varied diet from Europe since the end of isolationism, might have led to changes in the oral microbiome.

In the analysis of bacterial composition using next-generation sequencers, changes in the bacterial composition can occur due to differences in the types of primers, reference databases, and the database version, and reanalysis is recommended in the case of obtaining data from the database (Abellan-Schneyder et al., 2021; Abusleme et al., 2021). In this study, we reanalyzed the data obtained from the database to reduce the bias of the reference database and its version. In this study, 192 species were detected in the modern samples with periodontitis. There is a significant difference in the number of bacterial species detected between ancient and modern samples. One of the reasons is that in the modern samples, the V3–V4 region of the bacterial 16S rRNA gene was sequenced. According to Abellan-Schneyder et al. (2021), modern gut microbiota research using nine different primers (V1–V2, V1–V3, V3–V4, V4, V4–V5, V6–V8, and V7–V9) showed that the V3–V4 region primer had good overall performance. Furthermore, the V4 region should be included in the primer design since the V4 region was the best sub-region for phylogenetic resolution among other 16S rRNA gene variable regions (Yang et al., 2016). In modern research for bacterial composition in the oral cavity, many reports using the V3–V4 region have been performed, and good results have been reported (Maruyama et al., 2014; Komatsu et al., 2020; Shiba et al., 2021; Shimogishi et al., 2021). In contrast, many studies have shown effective results using ancient microbiome through the amplicon sequencing of the V4 region (Santiago-Rodriguez et al., 2017; Santiago-Rodriguez et al., 2019) because ancient DNA is known to be highly fragmented (Sawyer et al., 2012). Dietary patterns and socioeconomic factors can influence oral bacterial composition (Sawyer et al., 2012; Balakrishnan et al., 2021). Therefore, in this study, we focused on the sequence reads of modern samples from only studies conducted in Japan. Unfortunately, there have been no reports in Japan on analysis for the bacterial composition of subgingival plaque using the V4 region or amplicon sequencing of mixed supragingival and subgingival samples regardless of the kinds of primers used. For these reasons, we utilized the sequence data from DRA008582 (Iwauchi et al., 2019) and DRA010104 (Komatsu et al., 2020). The second reason is that unclassified bacteria accounted for a large percentage of the bacterial composition of Edo samples. This is thought to be the effect of low-quality ancient DNA and the lack of appropriate databases and analysis methods exclusively for the ancient microbiome. Therefore, the number of bacterial species in Edo samples with periodontitis was low. Ziesemer et al. (2015) reported that shotgun metagenomic sequencing was superior to amplicon sequencing of 16S rRNA gene in the analysis of ancient microbiome because ancient DNA is known to be highly degraded and fragmented, potentially leading to biased results compared with well-preserved DNA, such as modern DNA. However, the number of 16S rDNA reads was 0.2% of the total shotgun sequencing reads (Santiago-Rodriguez et al., 2017). Shotgun metagenomic sequencing needs a larger quantity of sample and is also more expensive. Additionally, many studies have shown effective results using ancient microbiome through the amplicon sequencing of the V4 region (Santiago-Rodriguez et al., 2017; Santiago-Rodriguez et al., 2019). Furthermore, the V4 region was the best sub-region for phylogenetic resolution among other 16S rRNA gene variable regions (Yang et al., 2016). Since the co-occurrence network of modern samples with periodontitis is simpler than that of Edo samples with periodontitis, it is easy to identify species that are at the core of the bacterial composition of modern samples with periodontitis. The results of this study showed that *P. gingivalis* is a core species connected to other typical periodontal pathogens, such as *T. forsythia* and *T. denticola*, in the co-occurrence networks for modern samples with periodontitis. In contrast, no typical periodontal pathogen was found in the co-occurrence network of Edo samples with periodontitis. This may indicate that the presence of bacterial species with no known pathogenicity might have influenced periodontitis in the Edo era, or the uniqueness and complexity of the microbial network of Edo samples with periodontitis itself might have contributed to the development of periodontal disease, although the number of specimens was limited and the evaluated Edo and modern samples were obtained from calculus and dental plaque, respectively.

## Conclusions

The dental calculus can preserve bacterial DNA and reveal the microbial composition in ancient samples. The modern bacterial composition of the oral cavity with periodontitis might be different from that of the Edo era, suggesting that periodontal pathogenic bacteria and its co-occurrence relationships might have changed with time and environment. To the best of our knowledge, this is the first study to investigate the periodontal microbiome in Japanese Edo samples accompanied by periodontal diagnoses based on skeletons and their micro-computed tomography.

## Publisher’s Note

All claims expressed in this article are solely those of the authors and do not necessarily represent those of their affiliated organizations, or those of the publisher, the editors and the reviewers. Any product that may be evaluated in this article, or claim that may be made by its manufacturer, is not guaranteed or endorsed by the publisher.

## Data Availability Statement

The datasets generated for this study can be found in the DNA Data Bank of Japan (http://www.ddbj.nig.ac.jp/) with the following accession number: DRA011882.

## Author Contributions

TSh, KK, TSu, and RS performed the experiments, processed the sequence data, and wrote the first draft of the manuscript. AS, TNe, CK, TNa, YO, SK, YT, HK, and TI assisted with the experiments and reviewed the manuscript. TSu, CK, and HK performed the morphological examinations. SU, TW, and TI supervised the analyses and wrote the manuscript. All authors contributed to the article and approved the submitted version.

## Funding

This work was supported by the Japan Society for the Promotion of Science (17H06662, 19K19016, and 21K16987 to TSh and 18K12563, 20H01370 to RS).

## Conflict of Interest

The authors declare that the research was conducted in the absence of any commercial or financial relationships that could be construed as a potential conflict of interest.

## Publisher’s Note

All claims expressed in this article are solely those of the authors and do not necessarily represent those of their affiliated organizations, or those of the publisher, the editors and the reviewers. Any product that may be evaluated in this article, or claim that may be made by its manufacturer, is not guaranteed or endorsed by the publisher.

## References

[B1] Abellan-Schneyder I. Matchado M. S. Reitmeier S. Sommer A. Sewald Z. Baumbach J.. (2021). Primer, Pipelines, Parameters: Issues in 16S rRNA Gene Sequencing. mSphere 6 (1). doi: 10.1128/mSphere.01202-20 PMC8544895 33627512

[B2] Abusleme L. Hoare A. Hong B. Y. Diaz P. I. (2021). Microbial Signatures of Health, Gingivitis, and Periodontitis. Periodontol 2000 86 (1), 57–78. doi: 10.1111/prd.12362 33690899

[B3] Adler C. J. Dobney K. Weyrich L. S. Kaidonis J. Walker A. W. Haak W.. (2013). Sequencing Ancient Calcified Dental Plaque Shows Changes in Oral Microbiota With Dietary Shifts of the Neolithic and Industrial Revolutions. Nat. Genet. 45 (4), 450–455. doi: 10.1038/ng.2536 23416520 PMC3996550

[B4] Balakrishnan B. Selvaraju V. Chen J. Ayine P. Yang L. Ramesh Babu J.. (2021). Ethnic Variability Associating Gut and Oral Microbiome With Obesity in Children. Gut Microbes 13 (1), 1–15. doi: 10.1080/19490976.2021.1882926 PMC7894456 33596768

[B5] Braun X. Ritter L. Jervøe-Storm P. M. Frentzen M. (2014). Diagnostic Accuracy of CBCT for Periodontal Lesions. Clin. Oral. Investig. 18 (4), 1229–1236. doi: 10.1007/s00784-013-1106-0 24048949

[B6] Brown K. DeCoffe D. Molcan E. Gibson D. L. (2012). Diet-Induced Dysbiosis of the Intestinal Microbiota and the Effects on Immunity and Disease. Nutrients 4 (8), 1095–1119. doi: 10.3390/nu4081095 23016134 PMC3448089

[B7] Burt B. (2005). Position Paper: Epidemiology of Periodontal Diseases. J. Periodontol 76 (8), 1406–1419. doi: 10.1902/jop.2005.76.8.1406 16101377

[B8] Cano R. J. Rivera-Perez J. Toranzos G. A. Santiago-Rodriguez T. M. Narganes-Storde Y. M. Chanlatte-Baik L.. (2014). Paleomicrobiology: Revealing Fecal Microbiomes of Ancient Indigenous Cultures. PloS One 9 (9), e106833. doi: 10.1371/journal.pone.0106833 25207979 PMC4160228

[B9] Caufield P. W. Schön C. N. Saraithong P. Li Y. Argimón S. (2015). Oral Lactobacilli and Dental Caries: A Model for Niche Adaptation in Humans. J. Dent. Res. 94 (9 Suppl), 110s–118s. doi: 10.1177/0022034515576052 25758458 PMC4547204

[B10] Chen W. P. Chang S. H. Tang C. Y. Liou M. L. Tsai S. J. Lin Y. L. (2018). Composition Analysis and Feature Selection of the Oral Microbiota Associated With Periodontal Disease. BioMed. Res. Int. 2018, 3130607. doi: 10.1155/2018/3130607 30581850 PMC6276491

[B11] Chen T. Yu W. H. Izard J. Baranova O. V. Lakshmanan A. Dewhirst F. E. (2010). The Human Oral Microbiome Database: A Web Accessible Resource for Investigating Oral Microbe Taxonomic and Genomic Information. Database (Oxford) 2010, baq013. doi: 10.1093/database/baq013 20624719 PMC2911848

[B12] Clarke N. G. Carey S. E. Srikandi W. Hirsch R. S. Leppard P. I. (1986). Periodontal Disease in Ancient Populations. Am. J. Phys. Anthropol 71 (2), 173–183. doi: 10.1002/ajpa.1330710205 3541645

[B13] Clemente J. C. Pehrsson E. C. Blaser M. J. Sandhu K. Gao Z. Wang B.. (2015). The Microbiome of Uncontacted Amerindians. Sci. Adv. 1 (3). doi: 10.1126/sciadv.1500183 PMC4517851 26229982

[B14] Darveau R. P. (2010). Periodontitis: A Polymicrobial Disruption of Host Homeostasis. Nat. Rev. Microbiol. 8 (7), 481–490. doi: 10.1038/nrmicro2337 20514045

[B15] Duran-Pinedo A. E. Chen T. Teles R. Starr J. R. Wang X. Krishnan K.. (2014). Community-Wide Transcriptome of the Oral Microbiome in Subjects With and Without Periodontitis. Isme J. 8 (8), 1659–1672. doi: 10.1038/ismej.2014.23 24599074 PMC4817619

[B16] Edwin L. Cooper N. Y. (2004). Complementary and Alternative Approaches to Biomedicine (Boston: Springer).

[B17] Eisenhofer R. Kanzawa-Kiriyama H. Shinoda K.-I. Weyrich L. S. (2020). Investigating the Demographic History of Japan Using Ancient Oral Microbiota. Philos. Trans. R. Soc. B: Biol. Sci. 375 (1812), 20190578. doi: 10.1098/rstb.2019.0578 PMC7702792 33012223

[B18] Falsen E. Pascual C. Sjödén B. Ohlén M. Collins M. D. (1999). Phenotypic and Phylogenetic Characterization of a Novel Lactobacillus Species From Human Sources: Description of Lactobacillus Iners Sp. Nov. Int. J. Syst Evol Microbiol. 49 (1), 217–221. doi: 10.1099/00207713-49-1-217 10028266

[B19] Fan J. Caton J. G. (2018). Occlusal Trauma and Excessive Occlusal Forces: Narrative Review, Case Definitions, and Diagnostic Considerations. J. Periodontol 89 Suppl 1, S214–s222. doi: 10.1002/jper.16-0581 29926937

[B20] Fujita H. (2012). Kobyourigaku-Jiten [in Japanese] (Japan: Douseisha).

[B21] Hajishengallis G. Darveau R. P. Curtis M. A. (2012). The Keystone-Pathogen Hypothesis. Nat. Rev. Microbiol. 10 (10), 717–725. doi: 10.1038/nrmicro2873 22941505 PMC3498498

[B22] Hanada A. Kurogi T. Giang N. M. Yamada T. Kamimoto Y. Kiso Y.. (2014). Bacteria of the Candidate Phylum TM7 are Prevalent in Acidophilic Nitrifying Sequencing-Batch Reactors. Microbes Environ. 29 (4), 353–362. doi: 10.1264/jsme2.ME14052 25241805 PMC4262358

[B23] Iwauchi M. Horigome A. Ishikawa K. Mikuni A. Nakano M. Xiao J. Z.. (2019). Relationship Between Oral and Gut Microbiota in Elderly People. Immun. Inflammation Dis. 7 (3), 229–236. doi: 10.1002/iid3.266 PMC6688080 31305026

[B24] Jeng J. H. Chan C. P. Ho Y. S. Lan W. H. Hsieh C. C. Chang M. C. (1999). Effects of Butyrate and Propionate on the Adhesion, Growth, Cell Cycle Kinetics, and Protein Synthesis of Cultured Human Gingival Fibroblasts. J. Periodontol 70 (12), 1435–1442. doi: 10.1902/jop.1999.70.12.1435 10632518

[B25] Jeraldo P. Kalari K. Chen X. Bhavsar J. Mangalam A. White B.. (2014). IM-TORNADO: A Tool for Comparison of 16S Reads From Paired-End Libraries. PloS One 9 (12), e114804–e114804. doi: 10.1371/journal.pone.0114804 25506826 PMC4266640

[B26] Karlsson F. H. Tremaroli V. Nookaew I. Bergström G. Behre C. J. Fagerberg B.. (2013). Gut Metagenome in European Women With Normal, Impaired and Diabetic Glucose Control. Nature 498 (7452), 99–103. doi: 10.1038/nature12198 23719380

[B27] Kassab M. M. Cohen R. E. (2003). The Etiology and Prevalence of Gingival Recession. J. Am. Dent. Assoc. 134 (2), 220–225. doi: 10.14219/jada.archive.2003.0137 12636127

[B28] Kasuga Y. Ishihara K. Okuda K. (2000). Significance of Detection of Porphyromonas Gingivalis, Bacteroides Forsythus and Treponema Denticola in Periodontal Pockets. Bull. Tokyo Dental Coll. 41 (3), 109–117. doi: 10.2209/tdcpublication.41.109 11212582

[B29] Kazui T. Videen S. D. (1982). Foreign Relations During the Edo Period: Sakoku Reexamined. J. Japanese Stud. 8 (2), 283–306. doi: 10.2307/132341

[B30] Kerr N. (1988). A Method of Assessing Periodontal Status in Archaeologically Derived Skeletal Material. Int. J. Paleopathol 2, 67–78.

[B31] Kerr N. W. (1991). Prevalence and Natural History of Periodontal Disease in Scotland – The Mediaeval Period (900–1600 A. D.). J. Periodontal Res. 26 (4), 346–354. doi: 10.1111/j.1600-0765.1991.tb02073.x 1831502

[B32] Kinane D. F. Stathopoulou P. G. Papapanou P. N. (2017). Periodontal Diseases. Nat. Rev. Dis. Primers 3, 17038. doi: 10.1038/nrdp.2017.38 28805207

[B33] Kitagawa M. (1853). Morisada-Manko [in Japanese].

[B34] Komatsu K. Shiba T. Takeuchi Y. Watanabe T. Koyanagi T. Nemoto T.. (2020). Discriminating Microbial Community Structure Between Peri-Implantitis and Periodontitis With Integrated Metagenomic, Metatranscriptomic, and Network Analysis. Front. Cell. Infect Microbiol. 10:596490 (773). doi: 10.3389/fcimb.2020.596490 33425781 PMC7793907

[B35] Lamont R. J. Koo H. Hajishengallis G. (2018). The Oral Microbiota: Dynamic Communities and Host Interactions. Nat. Rev. Microbiol. 16 (12), 745–759. doi: 10.1038/s41579-018-0089-x 30301974 PMC6278837

[B36] Larsen C. S. (1995). Biological Changes in Human Populations With Agriculture. Annu. Rev. Anthropol 24 (1), 185–213. doi: 10.1146/annurev.an.24.100195.001153

[B37] Lavigne S. E. Molto J. E. (1995). System of Measurement of the Severity of Periodontal Disease in Past Populations. Int. J. Osteoarchaeol 5 (3), 265–273. doi: 10.1002/oa.1390050305

[B38] Liu B. Faller L. L. Klitgord N. Mazumdar V. Ghodsi M. Sommer D. D.. (2012). Deep Sequencing of the Oral Microbiome Reveals Signatures of Periodontal Disease. PloS One 7 (6), e37919. doi: 10.1371/journal.pone.0037919 22675498 PMC3366996

[B39] Lloyd-Price J. Mahurkar A. Rahnavard G. Crabtree J. Orvis J. Hall A. B.. (2017). Strains, Functions and Dynamics in the Expanded Human Microbiome Project. Nature 550 (7674), 61–66. doi: 10.1038/nature23889 28953883 PMC5831082

[B40] Lozano M. Subirà M. E. Aparicio J. Lorenzo C. Gómez-Merino G. (2013). Toothpicking and Periodontal Disease in a Neanderthal Specimen From Cova Foradà Site (Valencia, Spain). PloS One 8 (10), e76852–e76852. doi: 10.1371/journal.pone.0076852 24146934 PMC3797767

[B41] Maruyama N. Maruyama F. Takeuchi Y. Aikawa C. Izumi Y. Nakagawa I. (2014). Intraindividual Variation in Core Microbiota in Peri-Implantitis and Periodontitis. Sci. Rep. 4 (1):6602. doi: 10.1038/srep06602 25308100 PMC4194447

[B42] Milici M. Deng Z.-L. Tomasch J. Decelle J. Wos-Oxley M. L. Wang H.. (2016). Co-Occurrence Analysis of Microbial Taxa in the Atlantic Ocean Reveals High Connectivity in the Free-Living Bacterioplankton. Front. Microbiol. 7:649 (649). doi: 10.3389/fmicb.2016.00649 27199970 PMC4858663

[B43] Ministry of Health, L.a.W (2012) Ministerial Notification No. 430 of the Ministry of Health, Labour and Welfare. Available at: https://www.mhlw.go.jp/file/06-Seisakujouhou-10900000-Kenkoukyoku/0000047330.pdf (Accessed June 7 2021).

[B44] Miyazaki Y. (1967). Nogyo Zensyo [in Japanese] (Japan: Iwanami-bunko).

[B45] Moore W. E. C. Cato E. P. Holdeman L. V. (1971). Eubacterium Lentum (Eggerth) Prévot 1938: Emendation of Description and Designation of the Neotype Strain. Int. J. Syst Evol Microbiol. 21 (4), 299–303. doi: 10.1099/00207713-21-4-299

[B46] Oyamada J. Kitagawa Y. Hara M. Sakamoto J. Matsushita T. Tsurumoto T.. (2017). Sex Differences of Dental Pathology in Early Modern Samurai and Commoners at Kokura in Japan. Odontology 105 (3), 267–274. doi: 10.1007/s10266-016-0275-0 27853978

[B47] Petersen P. E. Bourgeois D. Ogawa H. Estupinan-Day S. Ndiaye C. (2005). The Global Burden of Oral Diseases and Risks to Oral Health. Bull. World Health Organ 83 (9), 661–669. 16211157 PMC2626328

[B48] Pihlstrom B. L. Michalowicz B. S. Johnson N. W. (2005). Periodontal Diseases. Lancet 366 (9499), 1809–1820. doi: 10.1016/s0140-6736(05)67728-8 16298220

[B49] Pöllänen M. T. Overman D. O. Salonen J. I. (1997). Bacterial Metabolites Sodium Butyrate and Propionate Inhibit Epithelial Cell Growth In Vitro. J. Periodontal Res. 32 (3), 326–334. doi: 10.1111/j.1600-0765.1997.tb00541.x 9138199

[B50] Qu Y. Yang C. Ren Q. Ma M. Dong C. Hashimoto K. (2017). Comparison of (R)-Ketamine and Lanicemine on Depression-Like Phenotype and Abnormal Composition of Gut Microbiota in a Social Defeat Stress Model. Sci. Rep. 7 (1), 15725. doi: 10.1038/s41598-017-16060-7 29147024 PMC5691133

[B51] Santiago-Rodriguez T. M. Fornaciari A. Fornaciari G. Luciani S. Marota I. Vercellotti G.. (2019). Commensal and Pathogenic Members of the Dental Calculus Microbiome of Badia Pozzeveri Individuals From the 11th to 19th Centuries. Genes (Basel) 10 (4). doi: 10.3390/genes10040299 PMC6523138 31013797

[B52] Santiago-Rodriguez T. M. Narganes-Storde Y. Chanlatte-Baik L. Toranzos G. A. Cano R. J. (2017). Insights of the Dental Calculi Microbiome of Pre-Columbian Inhabitants From Puerto Rico. PeerJ 5, e3277. doi: 10.7717/peerj.3277 PMC5417066 28480145

[B53] Saso A. Kondo O. (2019). Periodontal Disease in the Neolithic Jomon: Inter-Site Comparisons of Inland and Coastal Areas in Central Honshu, Japan. Anthropological Sci. 127 (1), 13–25. doi: 10.1537/ase.190113

[B54] Sawafuji R. Saso A. Suda W. Hattori M. Ueda S. (2020). Ancient DNA Analysis of Food Remains in Human Dental Calculus From the Edo Period, Japan. PloS One 15 (3), e0226654. doi: 10.1371/journal.pone.0226654 32130218 PMC7055813

[B55] Sawyer S. Krause J. Guschanski K. Savolainen V. Pääbo S. (2012). Temporal Patterns of Nucleotide Misincorporations and DNA Fragmentation in Ancient DNA. PloS One 7 (3), e34131. doi: 10.1371/journal.pone.0034131 22479540 PMC3316601

[B56] Schloss P. D. Westcott S. L. Ryabin T. Hall J. R. Hartmann M. Hollister E. B.. (2009). Introducing Mothur: Open-Source, Platform-Independent, Community-Supported Software for Describing and Comparing Microbial Communities. Appl. Environ. Microbiol. 75 (23), 7537–7541. doi: 10.1128/aem.01541-09 19801464 PMC2786419

[B57] Shiba T. Watanabe T. Kachi H. Koyanagi T. Maruyama N. Murase K.. (2016). Distinct Interacting Core Taxa in Co-Occurrence Networks Enable Discrimination of Polymicrobial Oral Diseases With Similar Symptoms. Sci. Rep. 6 (1):30997. doi: 10.1038/srep30997 27499042 PMC4976368

[B58] Shiba T. Watanabe T. Komatsu K. Koyanagi T. Nemoto T. Ohsugi Y.. (2021). Non-Surgical Treatment for Periodontitis and Peri-Implantitis: Longitudinal Clinical and Bacteriological Findings-A Case Report With a 7-Year Follow-Up Evaluation. SAGE Open Med. Case Rep. 9:2050313x211029154. doi: 10.1177/2050313x211029154 PMC8261847 34285805

[B59] Shimogishi M. Watanabe T. Shibasaki M. Shiba T. Komatsu K. Nemoto T.. (2021). Patient-Specific Establishment of Bacterial Composition Within the Peri-Implant Microbiota During the Earliest Weeks After Implant Uncovering. J. Periodontal Res. doi: 10.1111/jre.12898 34057208

[B60] Smoot M. E. Ono K. Ruscheinski J. Wang P.-L. Ideker T. (2011). Cytoscape 2.8: New Features for Data Integration and Network Visualization. Bioinformatics 27 (3), 431–432. doi: 10.1093/bioinformatics/btq675 21149340 PMC3031041

[B61] Socransky S. S. Haffajee A. D. Cugini M. A. Smith C. Kent R. L. Jr. (1998). Microbial Complexes in Subgingival Plaque. J. Clin. Periodontol 25 (2), 134–144. doi: 10.1111/j.1600-051x.1998.tb02419.x 9495612

[B62] Takeuchi Y. Umeda M. Sakamoto M. Benno Y. Huang Y. Ishikawa I. (2001). Treponema Socranskii, Treponema Denticola, and Porphyromonas Gingivalis are Associated With Severity of Periodontal Tissue Destruction. J. Periodontol 72 (10), 1354–1363. doi: 10.1902/jop.2001.72.10.1354 11699477

[B63] Tse C. S. Williams D. M. (1992). Inhibition of Human Endothelial Cell Proliferation In Vitro in Response to N-Butyrate and Propionate. J. Periodontal Res. 27 (5), 506–510. doi: 10.1111/j.1600-0765.1992.tb01824.x 1403579

[B64] Uematsu H. Sato N. Hossain M. Z. Ikeda T. Hoshino E. (2003). Degradation of Arginine and Other Amino Acids by Butyrate-Producing Asaccharolytic Anaerobic Gram-Positive Rods in Periodontal Pockets. Arch. Oral. Biol. 48 (6), 423–429. doi: 10.1016/S0003-9969(03)00031-1 12749914

[B65] Ugarte-Torres A. Gillrie M. R. Griener T. P. Church D. L. (2018). Eggerthella Lenta Bloodstream Infections Are Associated With Increased Mortality Following Empiric Piperacillin-Tazobactam (TZP) Monotherapy: A Population-Based Cohort Study. Clin. Infect. Dis. 67 (2), 221–228. doi: 10.1093/cid/ciy057 29373647

[B66] van Houdt R. Ma B. Bruisten S. M. Speksnijder A. Ravel J. de Vries H. J. C. (2018). Lactobacillus Iners-Dominated Vaginal Microbiota is Associated With Increased Susceptibility to Chlamydia Trachomatis Infection in Dutch Women: A Case-Control Study. Sex Transm Infect. 94 (2), 117–123. doi: 10.1136/sextrans-2017-053133 28947665 PMC6083440

[B67] Velsko I. M. Fellows Yates J. A. Aron F. Hagan R. W. Frantz L. A. F. Loe L.. (2019). Microbial Differences Between Dental Plaque and Historic Dental Calculus are Related to Oral Biofilm Maturation Stage. Microbiome 7 (1), 102. doi: 10.1186/s40168-019-0717-3 31279340 PMC6612086

[B68] Warinner C. Rodrigues J. F. M. Vyas R. Trachsel C. Shved N. Grossmann J.. (2014). Pathogens and Host Immunity in the Ancient Human Oral Cavity. Nat. Genet. 46 (4), 336–344. doi: 10.1038/ng.2906 24562188 PMC3969750

[B69] Warinner C. Speller C. Collins M. J. (2015). A New Era in Palaeomicrobiology: Prospects for Ancient Dental Calculus as a Long-Term Record of the Human Oral Microbiome. Philos. Trans. R. Soc. London Ser. B Biol. Sci. 370 (1660), 20130376–20130376. doi: 10.1098/rstb.2013.0376 25487328 PMC4275884

[B70] Weyrich L. S. Duchene S. Soubrier J. Arriola L. Llamas B. Breen J.. (2017). Neanderthal Behaviour, Diet, and Disease Inferred From Ancient DNA in Dental Calculus. Nature 544 (7650), 357–361. doi: 10.1038/nature21674 28273061

[B71] Yang B. Wang Y. Qian P. Y. (2016). Sensitivity and Correlation of Hypervariable Regions in 16S rRNA Genes in Phylogenetic Analysis. BMC Bioinf. 17, 135. doi: 10.1186/s12859-016-0992-y PMC4802574 27000765

[B72] Yost S. Duran-Pinedo A. E. Teles R. Krishnan K. Frias-Lopez J. (2015). Functional Signatures of Oral Dysbiosis During Periodontitis Progression Revealed by Microbial Metatranscriptome Analysis. Genome Med. 7 (1), 27. doi: 10.1186/s13073-015-0153-3 25918553 PMC4410737

[B73] Ziesemer K. A. Mann A. E. Sankaranarayanan K. Schroeder H. Ozga A. T. Brandt B. W.. (2015). Intrinsic Challenges in Ancient Microbiome Reconstruction Using 16S rRNA Gene Amplification. Sci. Rep. 5 (1):16498. doi: 10.1038/srep16498 26563586 PMC4643231

