# Corrigendum: Comparison of Periodontal Bacteria of Edo and Modern Periods Using Novel Diagnostic Approach for Periodontitis With Micro-CT

**DOI:** 10.3389/fcimb.2022.871340

**Published:** 2022-04-13

**Authors:** Takahiko Shiba, Keiji Komatsu, Takeaki Sudo, Rikai Sawafuji, Aiko Saso, Shintaroh Ueda, Takayasu Watanabe, Takashi Nemoto, Chihiro Kano, Takahiko Nagai, Yujin Ohsugi, Sayaka Katagiri, Yasuo Takeuchi, Hiroaki Kobayashi, Takanori Iwata

**Affiliations:** ^1^Department of Periodontology, Graduate School of Medical and Dental Sciences, Tokyo Medical and Dental University, Tokyo, Japan; ^2^Department of Lifetime Oral Health Care Sciences, Graduate School of Medical and Dental Sciences, Tokyo Medical and Dental University, Tokyo, Japan; ^3^Institute of Education, Tokyo Medical and Dental University, Tokyo, Japan; ^4^The Graduate University for Advanced Studies, School of Advanced Sciences, Kanagawa, Japan; ^5^Department of Physical Therapy, Faculty of Rehabilitation, Niigata University of Health and Welfare, Kita-ku, Niigata, Japan; ^6^Department of Biological Sciences, Graduate School of Science, The University of Tokyo, Tokyo, Japan; ^7^Department of Legal Medicine, Toho University School of Medicine, Tokyo, Japan; ^8^Department of Chemistry, Nihon University School of Dentistry, Tokyo, Japan

**Keywords:** periodontitis, periodontal microbiome, edo era, 16S rDNA sequencing, ancient skeleton

## Error in Figure/Table

Error in Figures

In the original article, there were mistakes in **Figures 2** and **4** as published. The DRA number for modern samples without periodontitis in **Figures 2** and **4** was incorrect. The correct number is DRA008582. The corrected **Figures 2** and **4** appear below. The authors apologize for this error and state that this does not change the scientific conclusions of the article in any way.

**Figure 2:**
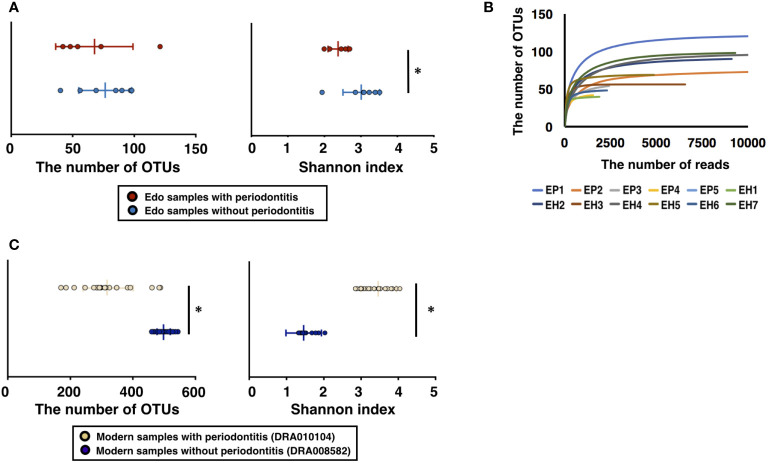
Evaluation of bacterial diversity of ancient Edo sample based on 16S rDNA sequences. **(A)** The number of operational taxonomic units (OTUs) and Shannon index of Edo samples. **(B)** Rarefaction curve of Edo samples. **(C)** The number of OTUs and Shannon index of modern samples. *P < 0.05.

**Figure 4:**
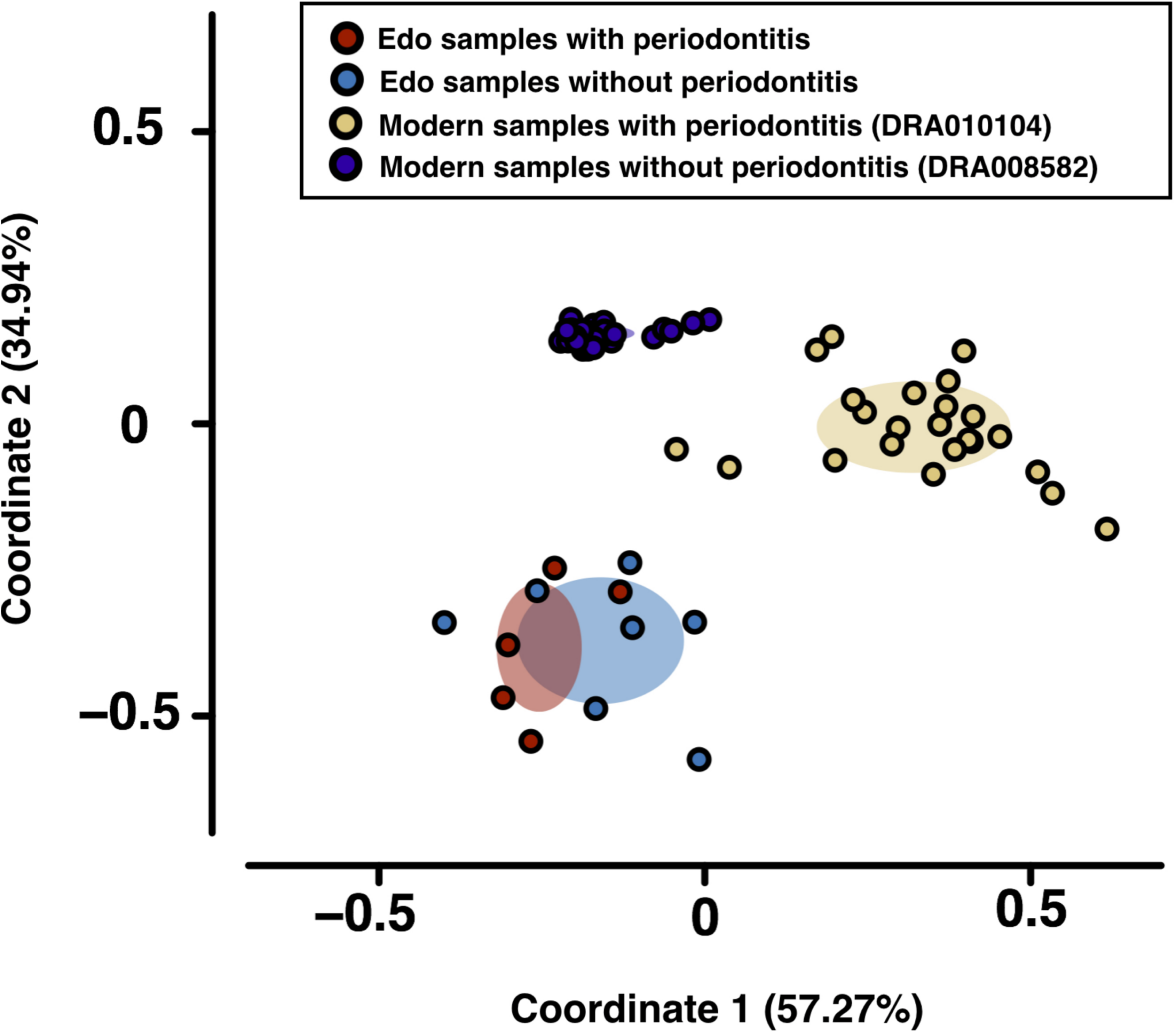
Principal coordinate analysis (PCoA) was conducted for the dissimilarity matrix value of 1—Spearman’s coefficient. PCoA was used to evaluate bacterial similarity for comparison between Edo and modern microbiomes according to class.

## Text Correction

In the original article, there was an error. The original article stated that “DRA012487” in the section of Data Availability Statement. This has been corrected to “DRA011882”. The corrected Data Availability Statement appears below.

The datasets generated for this study can be found in the DNA Data Bank of Japan (http://www.ddbj.nig.ac.jp/) with the following accession number: DRA011882.

The authors apologize for the error and state that it does not change the scientific conclusions of the article in any way. The original article has been updated.

## Publisher’s Note

All claims expressed in this article are solely those of the authors and do not necessarily represent those of their affiliated organizations, or those of the publisher, the editors and the reviewers. Any product that may be evaluated in this article, or claim that may be made by its manufacturer, is not guaranteed or endorsed by the publisher.

