# Supplementary material for: Comparison of Periodontal Bacteria of Edo and Modern Periods Using Novel Diagnostic Approach for Periodontitis With Micro-CT
Source: Front Cell Infect Microbiol. 2021 Sep 20;11:723821. doi: 10.3389/fcimb.2021.723821 (PMC8488429; doi:10.3389/fcimb.2021.723821)
Supplement: Supplementary file 1 [file Image_1.pdf]

Supplementary Material

Supplementary Figures

EP1

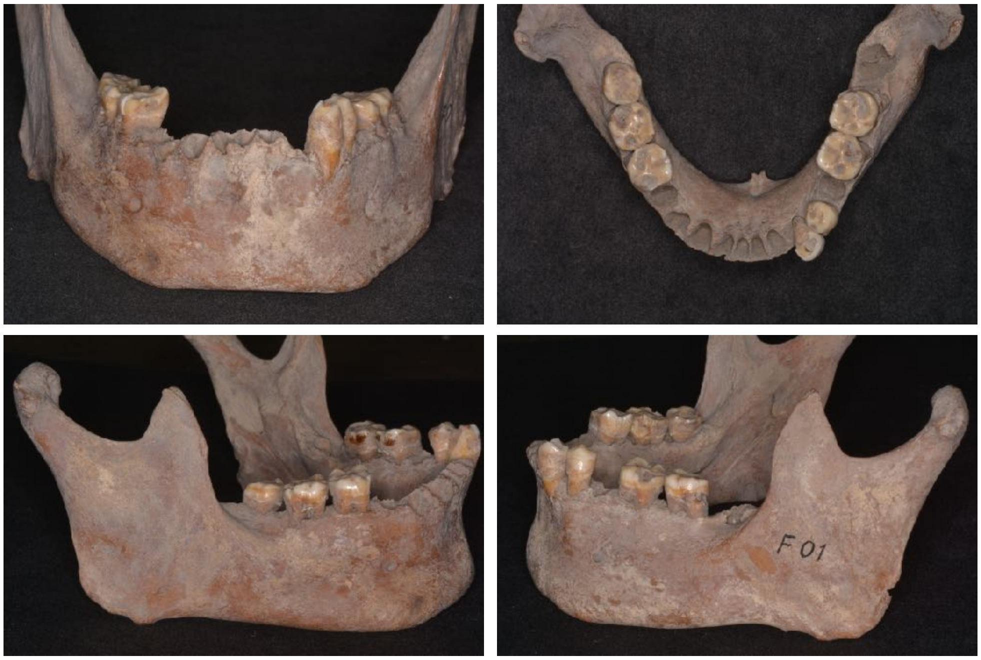

EP2

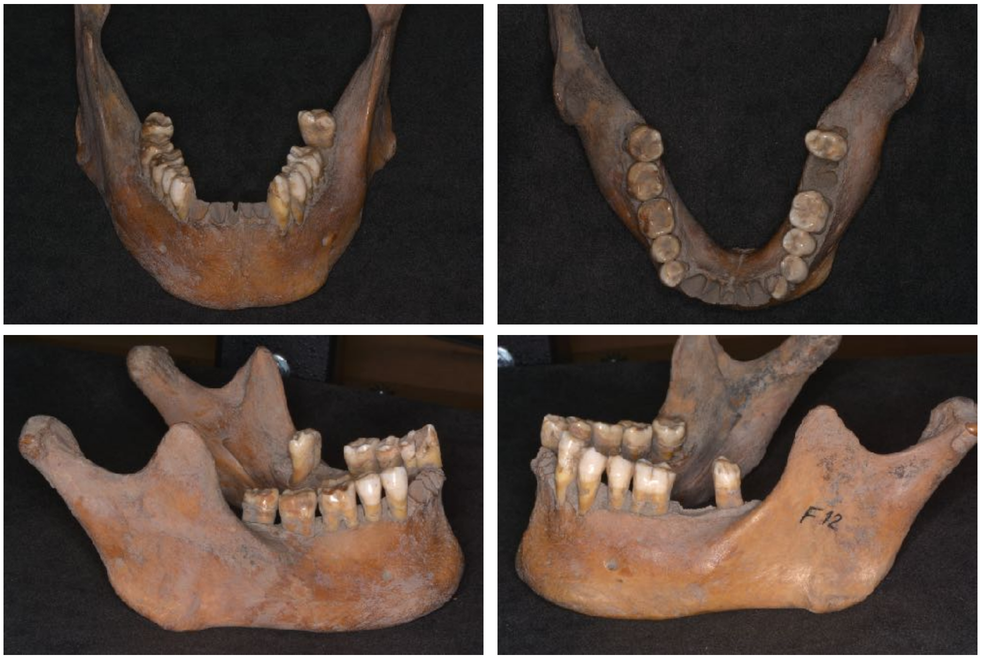

EP3

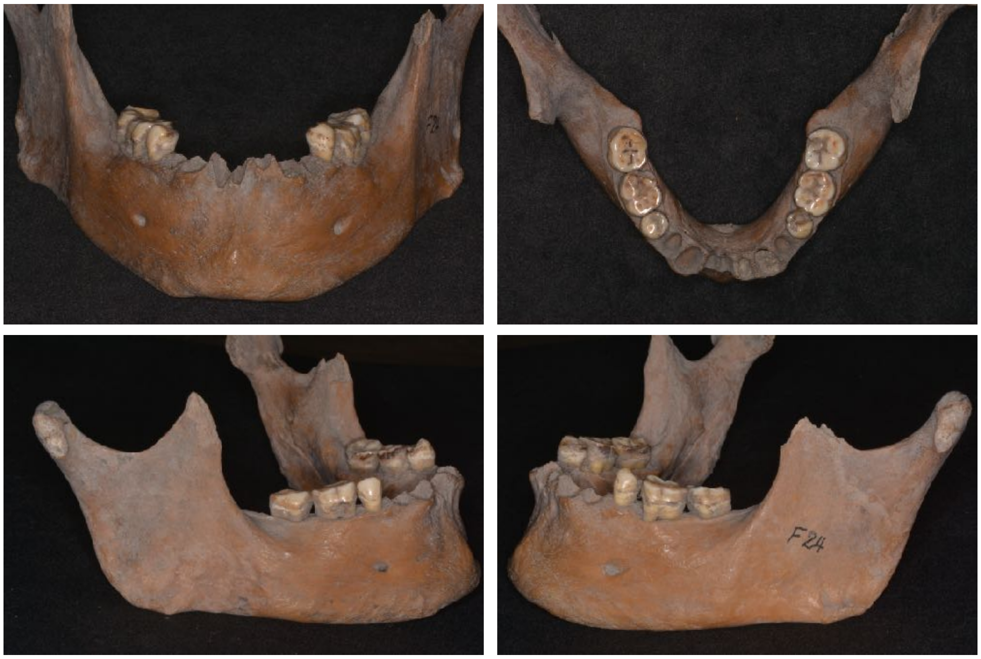

EP4

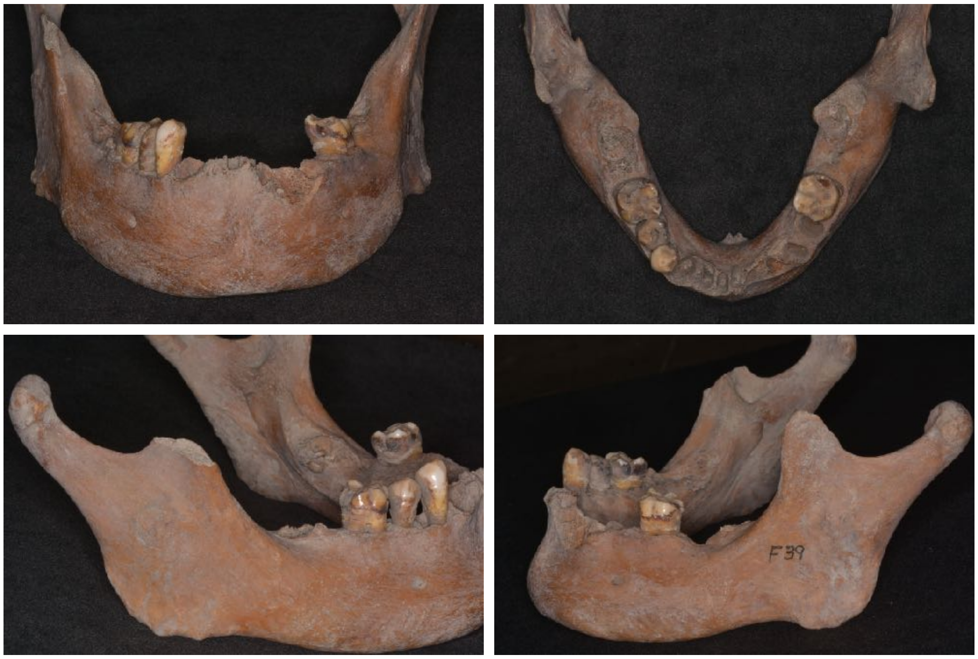

EP5

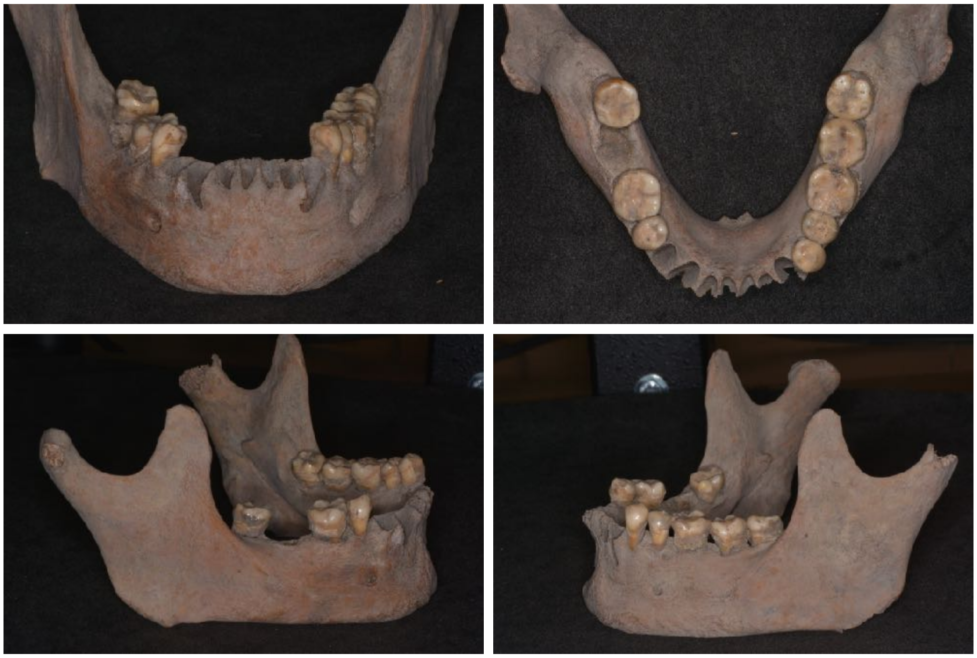

EH1

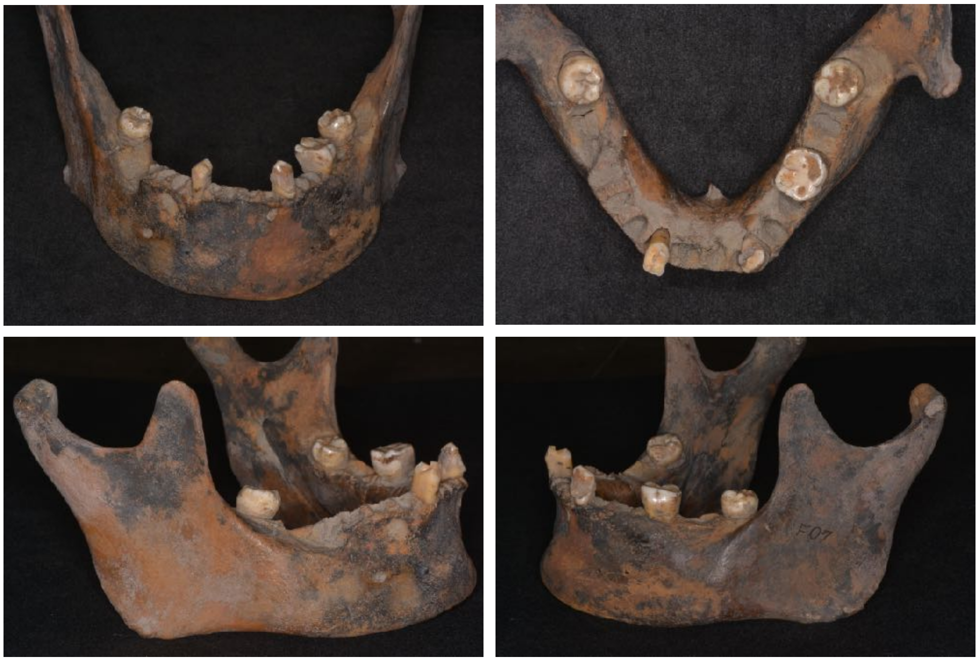

EH2

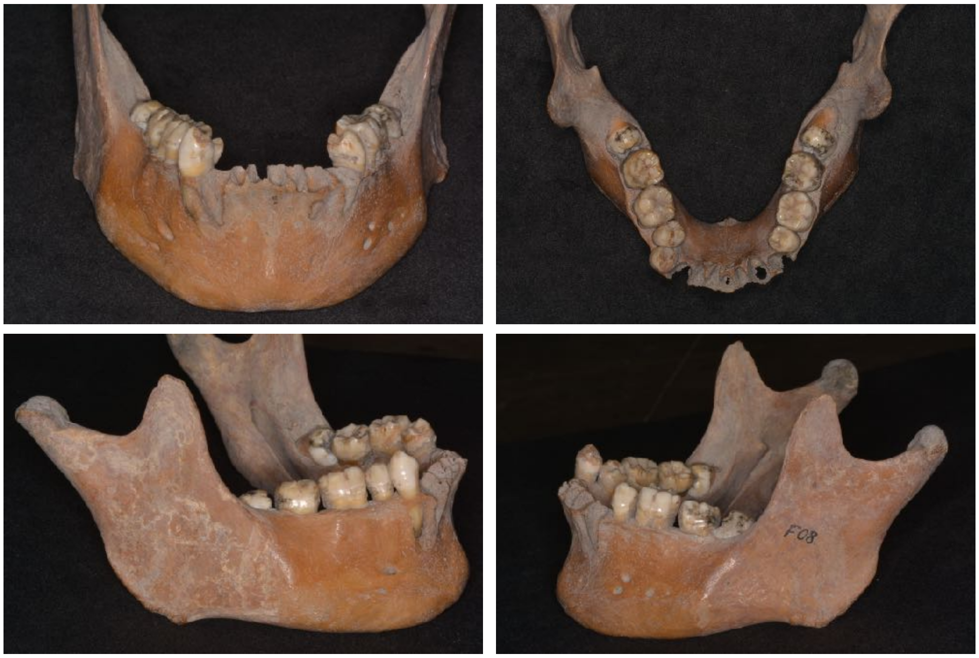

EH3

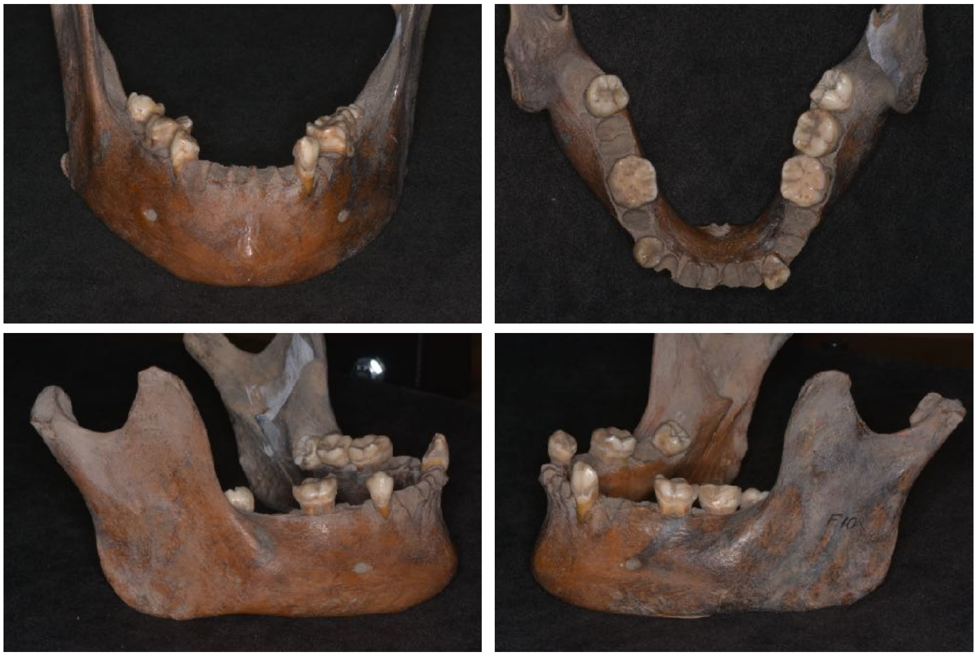

EH4

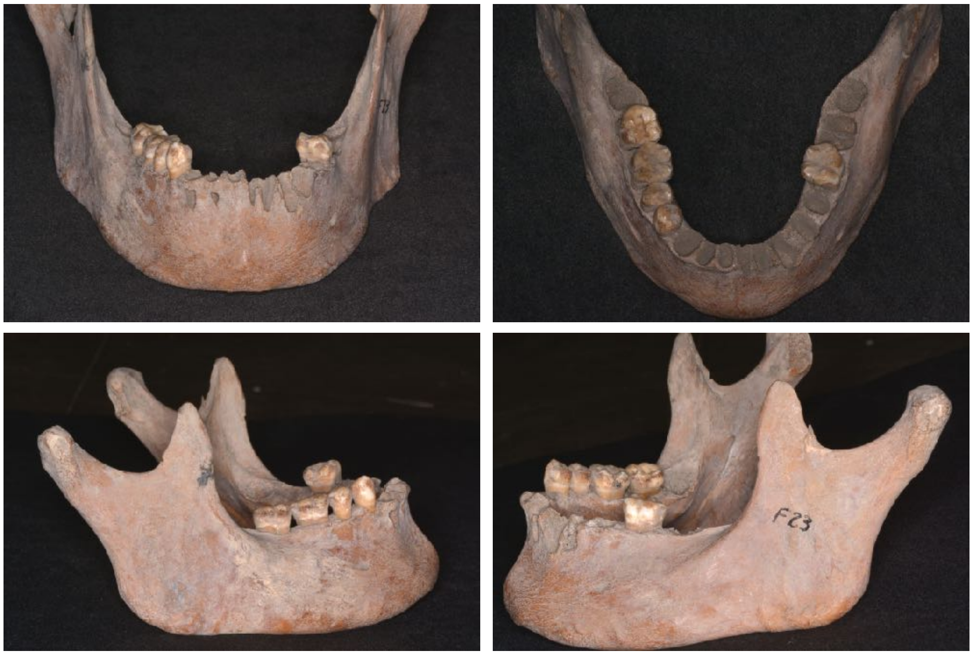

EH5

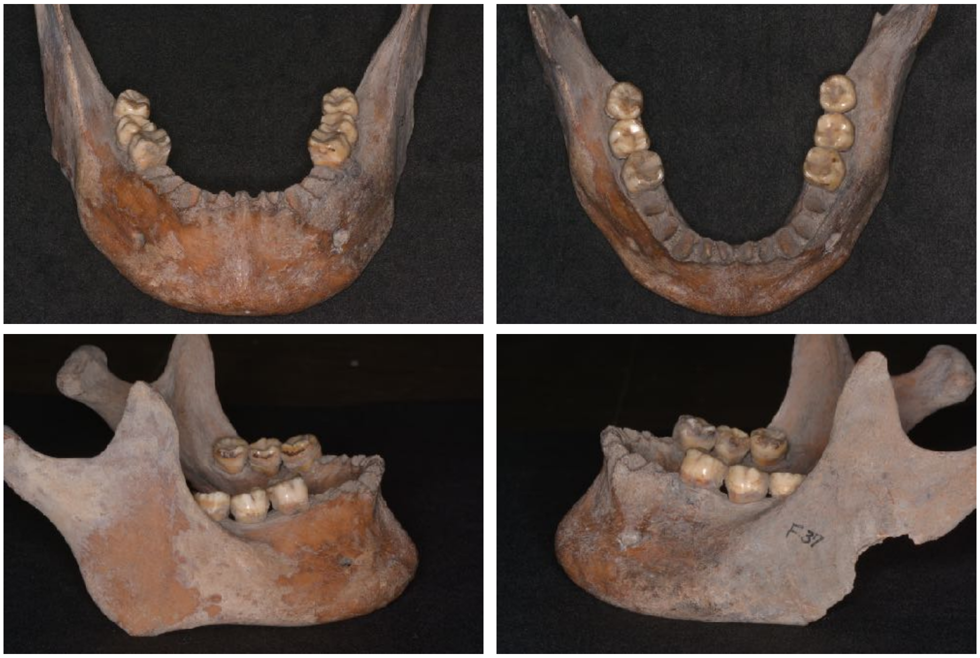

EH6

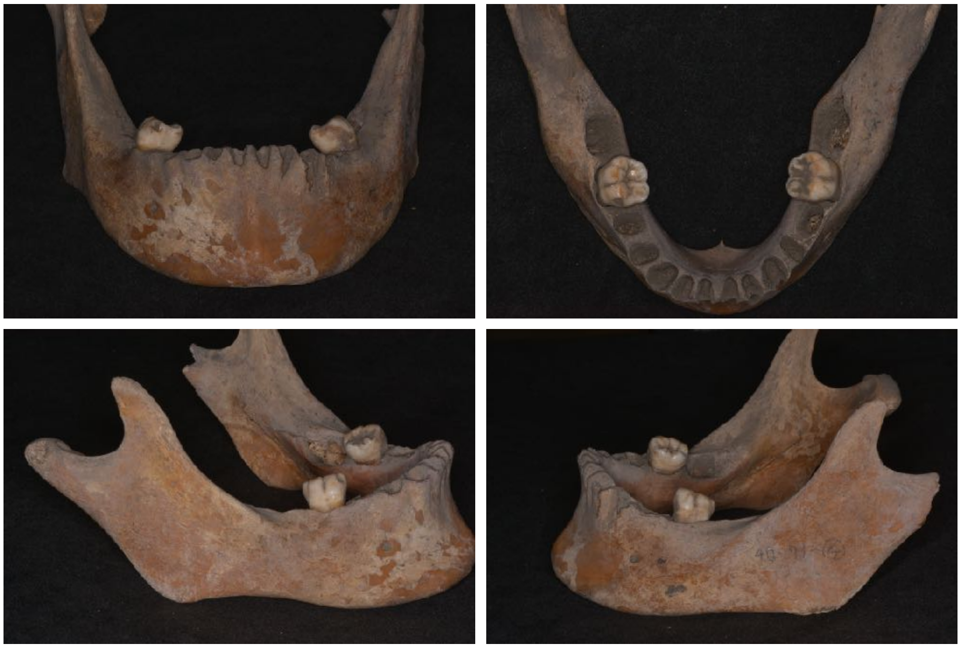

EH7

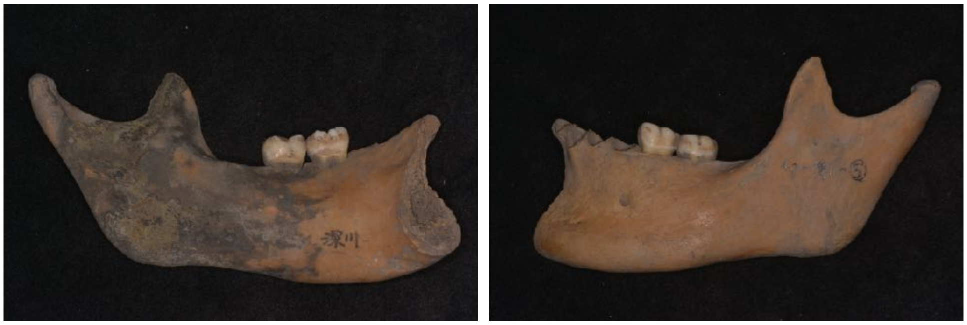

Only two photographs (lateral sides) exist in one sample (ID: EH7) because of the half of the jaw.

Supplementary Figure 1. Photographs of each skeleton. EP, Edo samples with periodontitis; EH, Edo sample without periodontitis. The sample number is consistent with that in Table 1.
